# Ultrapotent neutralizing antibodies against SARS-CoV-2 with a high degree of mutation resistance

**DOI:** 10.1172/JCI154987

**Published:** 2022-02-15

**Authors:** Jia Zou, Li Li, Peiyi Zheng, Wenhua Liang, Siyi Hu, Shuaixiang Zhou, Yanqun Wang, Jincun Zhao, Daopeng Yuan, Lu Liu, Dongdong Wu, Mengqiu Xu, Fangfang Zhang, Mengzhu Zhu, Zhihai Wu, Xiaochao Cao, Meng Ni, Xiaomin Ling, Yue Wu, Zhihui Kuang, Moyan Hu, Jianfeng Li, Xue Li, Xiling Guo, Tianmin Xu, Haiping Jiang, Changshou Gao, Michael Yu, Junjian Liu, Nanshan Zhong, Jianfeng Zhou, Jian-an Huang, Tengchuan Jin, Jianxing He

**Affiliations:** 1 Guangzhou Laboratory, Guangzhou International Biotech Island, Guangzhou, China.; 2 Innovent Biologics (Suzhou) Co., Ltd., Suzhou, Jiangsu, China.; 3 Hefei National Laboratory for Physical Sciences at Microscale, CAS Key Laboratory of Innate Immunity and Chronic Disease, School of Basic Medical Sciences, Division of Life Sciences and Medicine, University of Science and Technology of China, Hefei, China.; 4 State Key Laboratory of Respiratory Disease, the First Affiliated Hospital of Guangzhou Medical University, Guangzhou, China.; 5 Jiangsu Provincial Center for Disease Control and Prevention, Nanjing, China.; 6 Department of Infectious Diseases, The Third People’s Hospital of Changzhou, Changzhou, China.; 7 Department of Medical Oncology, First Affiliated Hospital, College of Medicine, Zhejiang University, Hangzhou, Zhejiang Province, China.; 8 Guangzhou Institute of Respiratory Health, Guangzhou, China.; 9 Department of Hematology, Tongji Hospital, Tongji Medical College, Huazhong University of Science and Technology, Wuhan, China.; 10 Department of Pulmonary and Critical Care Medicine, The First Affiliated Hospital of Soochow University, Suzhou, China.; 11 Department of Thoracic Oncology and Surgery, the First Affiliated Hospital of Guangzhou Medical University, Guangzhou, China.

**Keywords:** COVID-19, Drug screens, Drug therapy, Pharmacology

## Abstract

Many SARS-CoV-2 neutralizing antibodies (nAbs) lose potency against variants of concern. In this study, we developed 2 strategies to produce mutation-resistant antibodies. First, a yeast library expressing mutant receptor binding domains (RBDs) of the spike protein was utilized to screen for potent nAbs that are least susceptible to viral escape. Among the candidate antibodies, P5-22 displayed ultrahigh potency for virus neutralization as well as an outstanding mutation resistance profile. Additionally, P14-44 and P15-16 were recognized as mutation-resistant antibodies with broad betacoronavirus neutralization properties. P15-16 has only 1 binding hotspot, which is K378 in the RBD of SARS-CoV-2. The crystal structure of the P5-22, P14-44, and RBD ternary complex clarified the unique mechanisms that underlie the excellent mutation resistance profiles of these antibodies. Secondly, polymeric IgG enhanced antibody avidity by eliminating P5-22’s only hotspot, residue F486 in the RBD, thereby potently blocking cell entry by mutant viruses. Structural and functional analyses of antibodies screened using both potency assays and the yeast RBD library revealed rare, ultrapotent, mutation-resistant nAbs against SARS-CoV-2.

## Graphical abstract

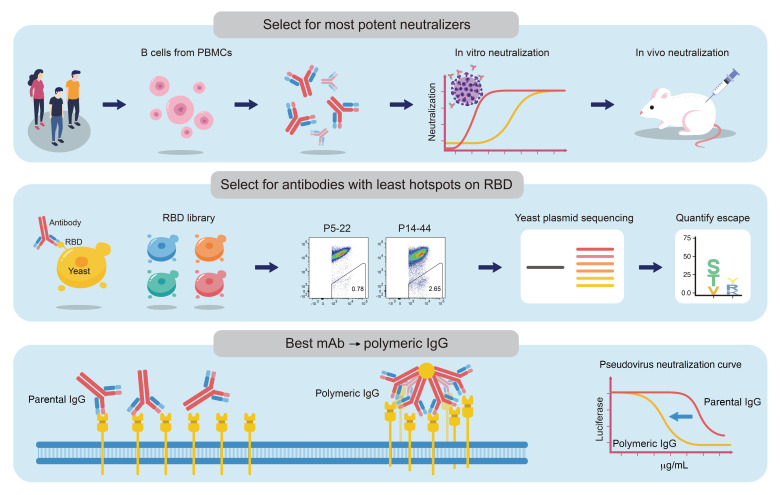

## Introduction

Neutralizing antibodies (nAbs), which show efficacy for prophylactic as well as therapeutic use, are being developed against the spike (S) protein of SARS-CoV-2 (1–8). These nAbs have shown efficacy in lowering the viral titer in COVID-19 patients and preventing them from developing severe COVID-19 (9–11). REGN-COV2, Eli Lilly’s cocktail (12, 13), and Vir/GSK’s sotrovimab have been approved for Emergency Use Authorization by the FDA. However, SARS-CoV-2, which is an RNA virus, manages to survive and spread by mutating (14). Although the mutation rate in coronaviruses is slower than that in most RNA viruses, a typical SARS-CoV-2 virus accumulates approximately 2 single-nucleotide mutations per month in its genome. The continuing transmission of SARS-CoV-2 in large populations worldwide has led to the evolution of variants of concern (VOCs) with increased transmissibility and possible resistance to current nAbs (15, 16). Considering that SARS-CoV-2 has only been spreading in the population for approximately 22 months without vaccine-induced antibody selection pressure, future viral escape of vaccine variants may pose a serious threat to attempts at ending the pandemic. Indeed, a recent discovery of a heavily mutated VOC, omicron, which has 32 mutations in the spike protein, reportedly overcomes Eli Lilly and Regeneron’s clinically developed cocktail and poses a serious threat to vaccine-elicited immunity. Thus, development of nAbs that can overcome existing and future viral mutation resistance is urgently needed. This necessitates the discovery of broad spectrum nAbs, such as ADG-2 and S309, that can block both SARS-CoV-1 and SARS-CoV-2 cell entry, in a manner that overcomes viral mutational escape as well (17, 18). However, mutations D405E, G502E/R/V, G504A/D/R/S/V, and Y505C/N/S were shown to abrogate ADG-2 binding (18), and S309 was also affected by viral mutations in the spike protein’s receptor binding domain (RBD) residues P337 and E340 (19, 20). Notably, the omicron variant has Y505H in its RBD. The presence of H505 has been demonstrated in the RBD of a closely related virus, RaTG13. Neither ADG-1 nor ADG-2 was able to bind to RaTG13’s RBD (18), suggesting a risk of these so-called broad nAbs in combatting omicron. Therefore, with the objective of overcoming current and future VOCs, we extensively screened SARS-CoV-2 nAbs obtained from dozens of convalescent donors to identify potent antibodies insensitive to amino acid substitutions in the RBD. Our efforts led to the discovery of potent nAbs capable of neutralizing authentic SARS-CoV-2 viruses from convalescent patients. More importantly, by testing the nAb’s ability to bind to a mutated-RBD yeast library, we identified several mutation-resistant antibodies. Notably, P5-22 and P15-16 have only 1 hotspot, which is unprecedented in reported nAbs so far. Consistent with this as well as the RBD–P5-22–P14-44 ternary crystal structure, a cocktail consisting of P5-22 plus P14-44, designated IBI314, did not lose potency against prevailing VOCs in pseudoviral neutralization and authentic-virus neutralization in vitro and showed protection at low doses in vivo. Finally, polymeric P5-22 with 12 antigen fragment binding regions (Fabs) was shown to have the greatest neutralization efficacy and prevent viral escape.

## Results

### Single B cell cloning of heavy and light chains from PBMCs of convalescent individuals.

**Figure 1:**
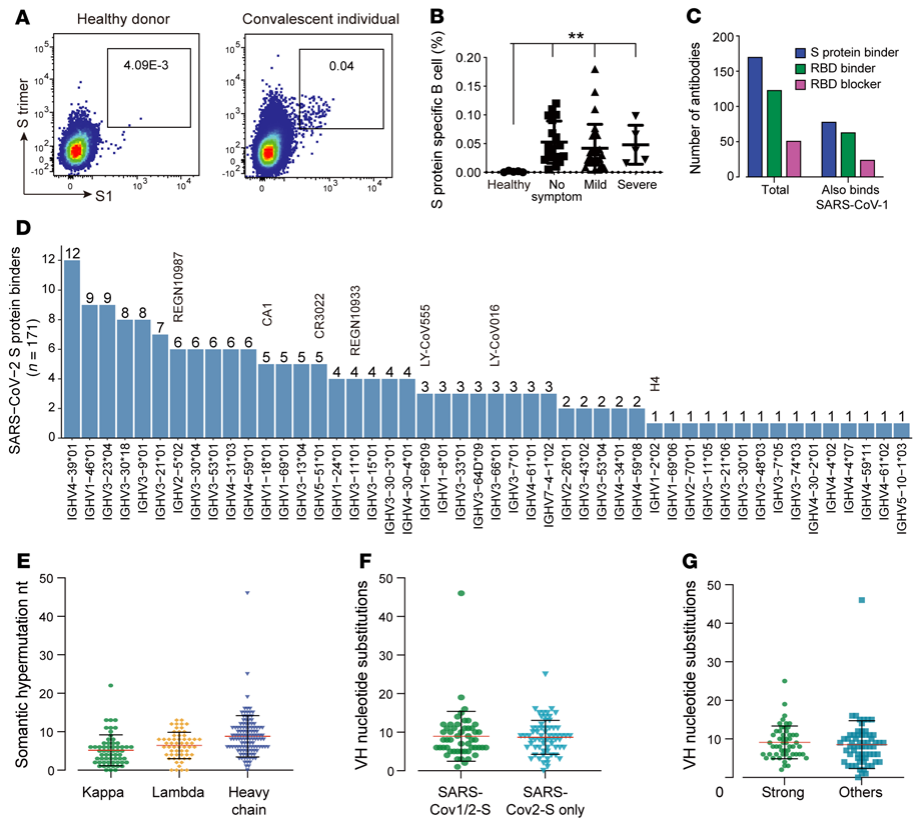
Neutralizing-antibody identification by single B cell cloning. (**A**) Representative FACS plots of gated B cells from healthy donors and convalescent donors stained using fluorescent spike (S) trimer and S1 subunit. (**B**) Statistics of S protein– and S1-specific B cell percentages of indicated donors. **P ≤ 0.01 by Student’s *t* test for differences between patient groups and healthy donor group. (**C**) Characteristics of single B cell cloned, and ELISA-validated antibodies that bind to SARS-CoV-2 or SARS-CoV-1 S/RBD proteins. (**D**) The distribution of IGHV gene usage for a total of 171 antibodies targeting the SARS-CoV-2 S protein. Reported nAbs using the corresponding IGHV genes are plotted. (**E**) Number of somatic nucleotide mutations in the IGHV and IGVL (κ or λ) in antibodies. (**F**) Number of somatic nucleotide mutations in the IGHV of SARS-CoV-1 and -2 S binders and SARS-CoV-2 S protein–only binders. (**G**) The number of somatic nucleotide mutations in IGHV of strong (*K_d_* < 3 × 10^–9^ M) or other S protein binders (*K_d_* > 3 × 10^–9^ M).

To isolate nAbs, we obtained 44 peripheral blood samples from convalescent patients who had recovered from COVID-19 for approximately 2 months, and isolated peripheral blood mononuclear cells (PBMCs) via Ficoll gradient centrifugation. Following B cell enrichment in PBMCs, we stained individual samples with a panel of B cell markers and fluorescently labeled SARS-CoV-2 S trimer protein and the S1 subunit. FACS analysis showed that after excluding plasma cells, the S1 and S trimer double-positive population within the B cells of healthy donors without SARS-CoV-2 exposure was approximately 0 to 0.003%. Although convalescent donors had substantially higher percentages of S1 and S trimer double-positive cells, ranging from 0.03% to 0.18%, there was no statistically significant difference between the S protein–reactive B cells of patients with different degrees of symptom severity (Figure 1, A and B). We isolated single B cells by FACS into 96 individual wells containing cell lysis buffer and an RNase inhibitor. Cognate antibody heavy chain and light chain pairs were obtained using reverse transcription PCR (RT-PCR) and sequencing. Two hundred forty pairs of heavy and light chains were cloned and expressed as human IgG1.

### Selection of RBD binders and blockers.

Among the 240 cloned antibodies, 171 were found to bind to the SARS-CoV-2 S protein, while 124 were found to bind to its RBD (Figure 1C). Interestingly, although our enrichment strategy used the SARS-CoV-2 S trimer and S1 proteins, 32.92% of S protein binders (79 antibodies) also bound to the SARS-CoV-1 S protein (Figure 1C), and 81.1% of those (64 antibodies) bound to the RBD of SARS-CoV-1 (Figure 1C and Supplemental Table 1; supplemental material available online with this article; https://doi.org/10.1172/JCI154987DS1). Competition ELISA showed that 41.94% of RBD binders could block the interaction between the SARS-CoV-2 RBD and human angiotensin-converting enzyme 2 (hACE2) (Figure 1C and Supplemental Table 1). Additionally, 31.65% of SARS-CoV-1 RBD binders (25 antibodies) also blocked the ACE2 interaction (Figure 1C and Supplemental Table 1). Sequence analysis indicated that all RBD blockers were unique, with no similar sequences in single B cell cloning (data not shown). A previous report showed that Ig variable region 3-53 (IGHV3-53) was the most frequently used region of the immunoglobulin heavy chain (*IGVH*) gene of RBD binders (21), but our spike binders showed a slightly different usage of IGHVs. In fact, IGHV4-39, IGHV1-46, IGHV3-23, IGHV3-30, IGHV3-9, and IGHV3-21 antibodies were present at a higher frequency than IGHV3-53 (Figure 1D and Supplemental Table 3). In addition, some potent nAbs reported and approved for clinical use were within the IGHV usage range (Figure 1D). Although we obtained the PBMC samples just 2 months after patient recovery, most of the S protein binders had 10 to 50 nt of somatic hypermutation in heavy and light chains, indicating some degree of affinity maturation (Figure 1E). A report based on convalescent patients’ antibody hypermutation levels indicated that the average antibody somatic hypermutation level we observed was between the levels of antibody-gene mutation found in B cells from individuals between 1.3 and 6.2 months after infection (22). This indicated that antibody somatic hypermutation levels would increase with time, indicating continuous affinity maturation in convalescent patients. There was no difference in somatic hypermutation between binders of SARS-CoV-1 and SARS-CoV-2 S proteins and SARS-CoV-2–only binders (Figure 1F). In addition, when binders with *K_d_* lower than 3 × 10^-9^ M were defined as strong binders, there was no correlation between somatic hypermutation and antibody affinity (Figure 1G). We concluded that SARS-CoV-2 infection may induce a variable frequency of B cells capable of secreting antibodies that are able to bind to the S protein, particularly the RBD domain. Notably, a significant percentage of SARS-CoV-2 S protein and RBD binders cross-react with their counterparts of SARS-CoV-1 without having to undergo somatic hypermutation.

### Selection of potent nAbs and epitope binning.

Next, we screened RBD binders for their ability to neutralize pseudovirus in vitro, and found 118 antibodies that showed more potent neutralization of SARS-CoV-2 pseudovirus in vitro than the benchmark antibodies, B38 (23), H4 (23), CA1, and CB6 (ref. 13 and Supplemental Figure 1). Following a developability assessment of critical quality attributes associated with chemical stability and undesirable posttranslational modifications, 49 antibodies were selected for the determination of epitope binning and IC_50_ (half maximal inhibitory concentration) values pertaining to pseudovirus neutralization (Supplemental Table 2). We also performed an epitope binning experiment that identified 6 distinct bins for antibody binding (Supplemental Figure 2, A and C, and Supplemental Table 2). In general, SARS-CoV-2 RBD–blocking antibodies with lower IC_50_ values showed superior pseudovirus neutralization activity, and occupied bins 1, 4, and 5 (Supplemental Table 2 and Supplemental Figure 3, A and C). Antibodies that are cross-blockers of SARS-CoV-1 or nonblockers of RBD/ACE2 in ELISAs showed less potency in the pseudovirus neutralization assay and occupied bins 2, 3, and 6 (Supplemental Table 2 and Supplemental Figure 3, B and C).

### Potent antibody cocktail neutralizes authentic SARS-CoV-2 viral infection in vitro and in vivo.

**Figure 2:**
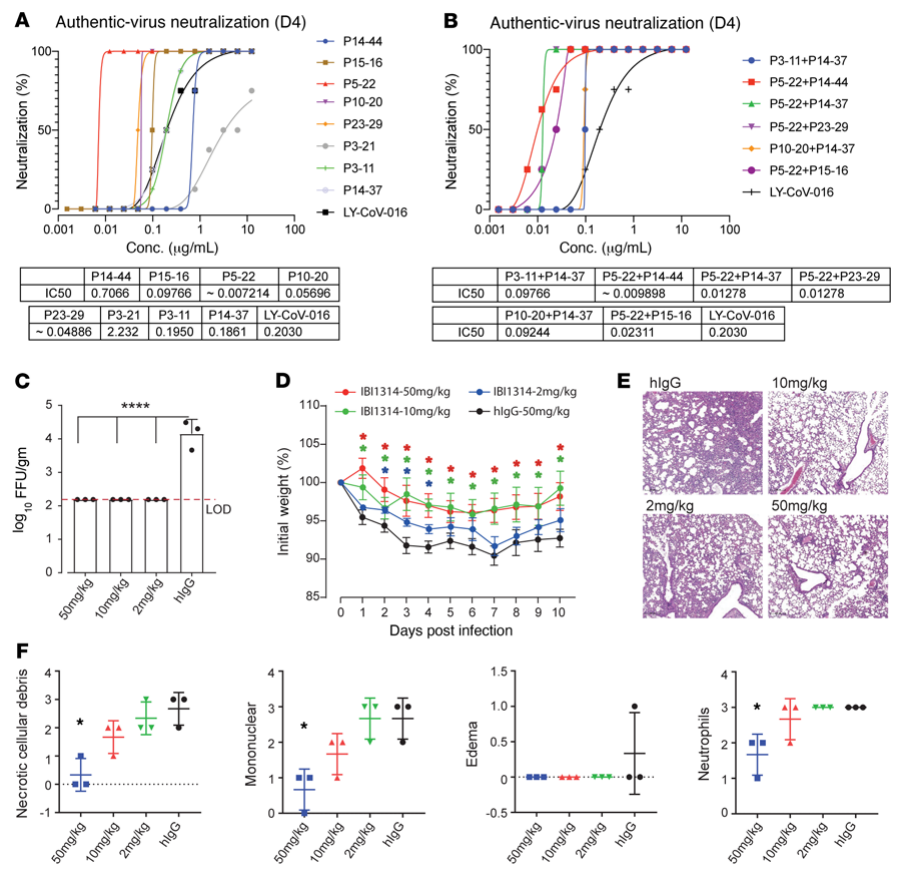
Selected potent antibodies protect against authentic SARS-CoV-2 infection in vitro and in vivo. (**A** and **B**) Authentic-virus neutralization assay using SARS-CoV-2 (BetaCoV/JS02/human/2020) to evaluate monoclonal antibodies (**A**) and monoclonal antibody cocktails (**B**) in vitro. (**C**) Ad5-hACE2–transduced mice were infected with SARS-CoV-2/human/CHN/IQTC01/2020 (NCBI accession number MT123290) and treated with different doses of IBI314 (1 dpi, i.p.), and lungs were harvested to measure viral titers 3 dpi (*n =* 3 mice per group). Multiple comparisons were performed by 1-way ANOVA. ****P ≤ 0.0001. (**D**) Daily mouse body weight (*n =* 5 mice per group) and (**E**) sections of paraffin-embedded lungs were stained with hematoxylin and eosin on dpi 4 (*n =* 3 mice per group). Scale bars: 200 μm. (**F**) Summary of histological scores in **E**. *P ≤ 0.05 by 1-tailed Student’s *t* test (**D** and **F**). Data are represented as mean ± SD.

Finally, we selected P3-11, P5-22, P10-20, P14-37, P14-44, P15-16, P23-29, and P3-21, which exhibited potent pseudovirus neutralization with distinct epitope binding, as well as authentic-virus neutralization in vitro. The IC_50_ values obtained via the authentic-virus neutralization assay indicated that all tested monoclonal antibodies displayed a potency equivalent to, or significantly more potent than, that of LY-CoV016, except for the non–RBD/ACE2-blocking antibody, P3-21 (Figure 2A). Notably, P5-22 showed ultrapotent authentic-virus neutralization activity, with an IC_50_ value that was only 1/30 of that of LY-CoV016 (Figure 2A). Based on epitope binning information, we constructed a 1:1 cocktail of potent nAbs, which also showed ultrapotent neutralization of SARS-CoV-2 (Figure 2B). P5-22/P14-44 was the most potent of the 1:1 combination of 2-antibody cocktails that were formulated (Figure 2B). Experimental results indicated that P5-22 bound tightly to the SARS-CoV-2 S RBD with an affinity higher than 3.34 pM, owing to the extremely slow off rate (Supplemental Figure 4A). Furthermore, P14-44 showed a high affinity for both the SARS-CoV-2 S protein (0.55 nM) and for SARS-CoV-1 S protein (4.3 nM) (Supplemental Figure 4B). Thus, this P5-22/P14-44 cocktail was designated as IBI314 and subjected to an in vivo study. The therapeutic efficacy of IBI314 was evaluated in vivo using SARS-CoV-2–infected Ad5-hACE2–transduced mice, which have been validated as a mouse SARS-CoV-2 infection model by multiple studies (24–28). Ad5-hACE2–transduced BALB/c mice were treated with 2, 10, and 50 mg/kg IBI314, 1 day following SARS-CoV-2 infection. Lungs were harvested for virus titration at 3 days postinfection (dpi) (Figure 2C). IBI314 treatment led to a 2-log reduction in the lung viral titer on day 3, with undetectable levels in all mice. Evaluation of dose-dependent therapeutic effects showed that administering the IBI314 cocktail at doses higher than 2 mg/kg produced a stronger protective effect that reduced viral loads in the lungs (Figure 2C). Infected mice in the different dose-therapeutic groups showed significantly reduced weight loss, as compared with hIgG-treated control mice (Figure 2D). Lung sections were obtained at 4 dpi. Examination of lung tissues from SARS-CoV-2–infected mice demonstrated a variety of lesions, including perivascular to interstitial inflammatory cell infiltrates and necrotic cell debris. A dose of 50 mg/kg IBI314 cocktail prevented peribranchial lymphoid infiltration and bronchial epithelial cell damage, whereas control mice progressed to interstitial pneumonia (Figure 2, E and F). Lung viral titer and weight loss results indicated that IBI314 protected mice from SARS-CoV-2 infection. Treatment with the IBI314 cocktail did not cause viral mutations in vivo (Supplemental Figure 5).

### Selection for mutation-resistant antibodies.

**Figure 3:**
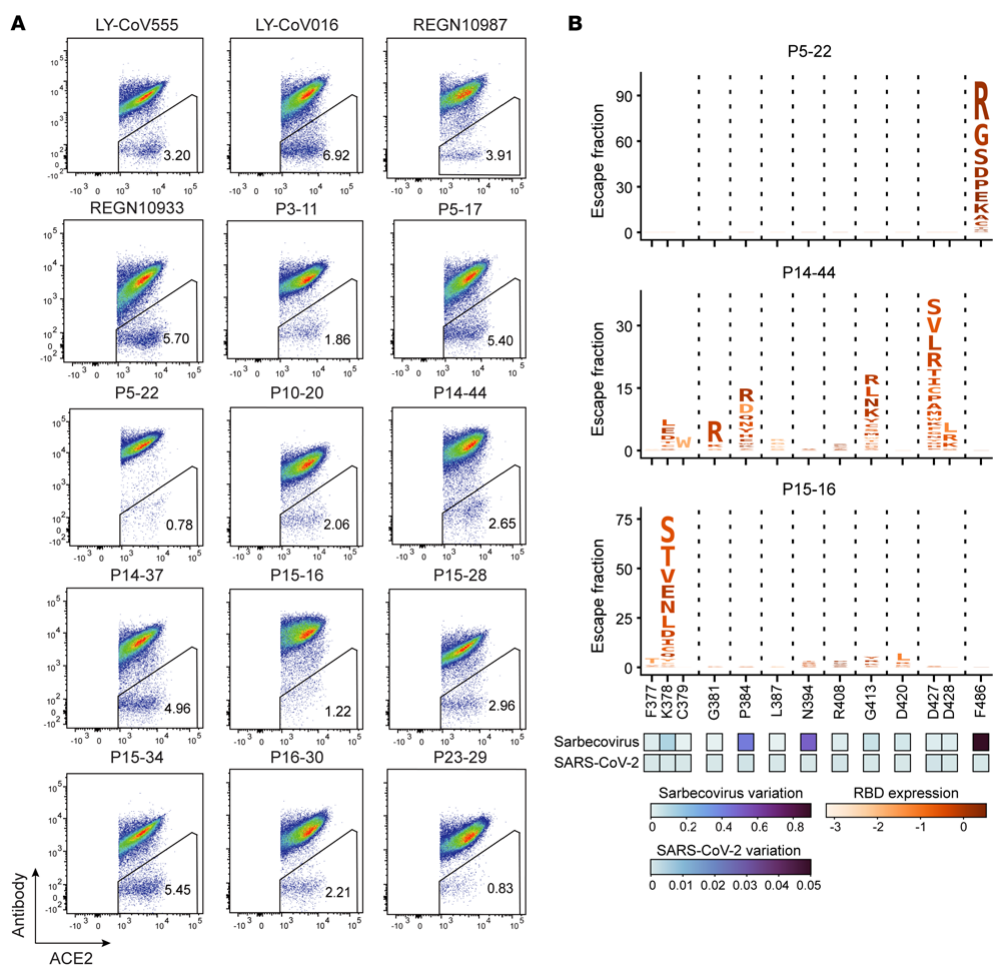
Yeast library displaying mutant RBDs showed superior resistance of viral mutation–escape antibodies. (**A**) FACS analysis of fluorescent SARS-CoV-2 nAbs and fluorescent hACE2 staining of yeast library displaying mutant RBDs; hACE2-binding cells are shown. (**B**) Logo plots showing mutational scanning maps of mutations that escape binding by P5-22, P14-44, and P15-16 targeting the RBD. Letter height indicates the escape fraction from antibody binding. Letters are colored according to effect on RBD ACE2 affinity and RBD expression (53). Reported mutations among SARS-CoV-2 sequences in GISAID and RBD sequence alignment among Sarbecoviruses were used to generate heatmaps to visualize mutations of each residue reflecting the likelihood of mutation occurrence.

In order to select antibodies that are resistant to viral escape, a yeast library that displayed mutant RBDs covering all possible single amino acid mutations over the entire RBD protein was constructed. Staining with fluorescently labeled anti-ACE2 and candidate antibodies enabled our candidate nAbs to be compared with 4 benchmark antibodies from Eli Lilly and Regeneron cocktails. Yeasts that bind to ACE2, but not to nAbs, were defined as escapees. Escapees from benchmark antibodies from Eli Lilly and Regeneron cocktails ranged from 3.2% to 6.9%, whereas only 2 of the 11 tested candidate antibodies fell within this range (Figure 3A). Notably, P5-22, P15-16, and P23-29 had less than 1.22% escapees (Figure 3A). We sorted the escapees from P5-22, P14-44, P15-16, and benchmark antibodies for plasmid sequencing. Approximately 300 reference antibody escapee plasmid samples were sequenced, although this was not sufficient for full coverage based on FACS percentages of escapees. The mutational landscape of the benchmark antibody group showed an escape profile that was very similar to that stated in recent reports (Supplemental Figure 6 and refs. 29–31). P5-22, P5-16, and P14-44 escapee samples were subjected to at least 5 times as many sequencing counts as FACS-selected yeast cells. Plasmid sequencing results showed excellent mutation-resistant properties of P5-22 and P15-16. P5-22 was susceptible to the F486 mutation (Figure 3B), and P15-16 was almost exclusively susceptible only to the K378 mutation (Figure 3B). Although P14-44 was susceptible to K378, G381, P384, D427/428, and G413 mutations (Figure 3B), these residues, except for P384A in SARS-CoV-1, are highly conserved in clade B betacoronaviruses, as illustrated by us (Supplemental Figure 13) and other reports (32, 33), indicating a possible functionally critical role for these residues in S proteins. Indeed, by data mining a database of all sequenced SARS-CoV-2 variants (GISAID database: 3,722,275 sequences), we found that these residues are virtually unmutated in the variant database (Figure 3B, heatmap). In contrast, the susceptible residues in LY-COV555, LY-COV-016, REGN10933, and REGN10987 contain the high-risk residues L452, E484, K417, and N439, which are more frequently mutated (Supplemental Figure 6, heatmap). Moreover, by examining the broad Sarbecovirus (the viral subgenus containing SARS-CoV-1 and SARS-CoV-2) sequences, most of the P14-44 hotspot residues are conserved, with low mutation frequency across virus species (Figure 3B). In sharp contrast, LY-COV555, LY-COV-016, REGN10933, and REGN10987 hotspot residues are mainly highly mutated, indicating these nAbs are poor broad neutralizers across species (Supplemental Figure 6). It is noteworthy that P14-44 was not susceptible to P384A; instead, it potently binds to the SARS-CoV-1 RBD and neutralizes the SARS-CoV-1 pseudovirus (Supplemental Figure 4 and Supplemental Figure 8, E and F).

### P5-22, P14-44, and P15-16 show no loss of neutralizing efficiency against current VOCs.

**Figure 4:**
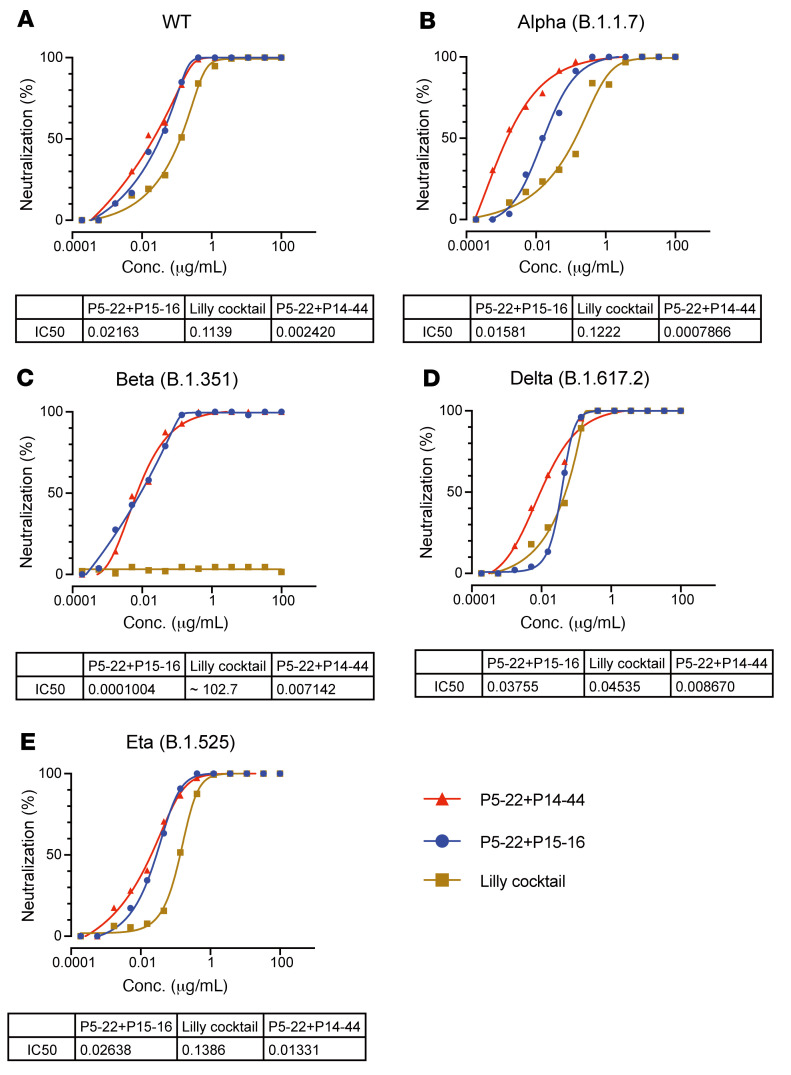
Neutralization potency of authentic live VOCs by IBI314 and IBI314 backup cocktail in vitro. (**A**–**D**) Authentic-virus neutralization curve of IBI314 (P5-22+P14-44) and its backup cocktail (P5-22+P15-16) in neutralizing WT (**A**), alpha mutant strain B.1.1.7 (**B**), beta mutant strain B.1.351 (**C**), delta mutant strain B.1.617.2 (**D**), and eta mutant strain B.1.525 (**E**).

To test the potential neutralizing efficiency of P5-22, P14-44, and P15-16 with mutated viruses, we first examined the affinity of these antibodies for either the WT RBD or a series of reported RBD mutants (E406W, F486V, K417V, N481L, S477A, N450L, Y508H, F456V, L455V, K417N/E484K/N501, K444Q, V445A, L452R, Y453F, V483A, F490L, N439K, N450D, L455F, A475V, E484Q, F490P, and Q493K), which escaped other nAbs. P5-22, P14-44, and P15-16 showed no reduction in binding to all these mutations except to F486V, which decreased P5-22 binding (Supplemental Figure 7). This is consistent with yeast library screening results. This indicated that our nAbs were resistant to natural variants. To investigate this, we tested our antibodies against B.1.1.7 (alpha), B.1.351 (beta), and B1.617.1 (kappa) pseudoviruses in a neutralization assay in vitro. Compared with the IC_50_ of WT, P5-22 and P14-44 showed no loss of potency against these VOCs (Supplemental Figure 8). Additionally, P14-44 and P15-16 more potently neutralized the SARS-CoV-1 pseudovirus than CR3022 and had comparable potency against S309 (Supplemental Figure 8, E and F), which are benchmark broad neutralizers (6, 34). Next, we isolated authentic SARS-CoV-2 variants and performed an in vitro neutralization assay with IBI314 (P5-22/P14-44), IBI314 backup cocktail (P5-22/P15-16), and Eli Lilly cocktail (LY-COV016/LY-COV555). The results showed that the IBI314 cocktail did not decrease the potency for neutralizing all VOCs in vitro (Figure 4, A–E). The IBI314 backup cocktail, P5-22/P15-16, also showed uncompromised potency, although its IC_50_ was slightly higher than that of IBI314 (Figure 4, A–E). By contrast, the official formulation (LY-COV016/LY-COV555 [2:1]; ref. 35) did not neutralize the beta strain at all (Figure 4C). It also showed much lower potency in neutralizing WT, alpha, and eta strains, compared with IBI314 (Figure 4, A, B, and E). Considered together, these results show that both IBI314 and its backup cocktail may potently block the entry of the pseudovirus as well as the authentic live SARS-CoV-2 mutant virus in vitro without compromising potency.

### Crystal structure of P5-22, P14-44, and RBD complex.

To provide further insight into the neutralizing mechanism and resistance to RBD mutation escape of P14-44 and P5-22 antibodies, a series of crystallization experiments were performed. The crystal structures of the Fabs of P14-44 and P5-22 complexes with the SARS-CoV-2 RBD and the P14-44 complex with SARS-CoV-2 RBD were determined at 2.4 Å and 1.9 Å resolution, respectively. Notably, there were no significant conformational changes in VH and VL in the binary and ternary complexes of P14-44, with overall root mean square deviations (RMSDs) of 0.154 Å and 0.149 Å, respectively (Supplemental Figure 10). The structure of the ternary complex demonstrates that 2 Fabs can simultaneously bind to distinct epitopes on the RBD, which is consistent with the result of the epitope binning assay that showed that these were noncompeting antibodies (Supplemental Figure 2B and Supplemental Figure 5A).

**Figure 5:**
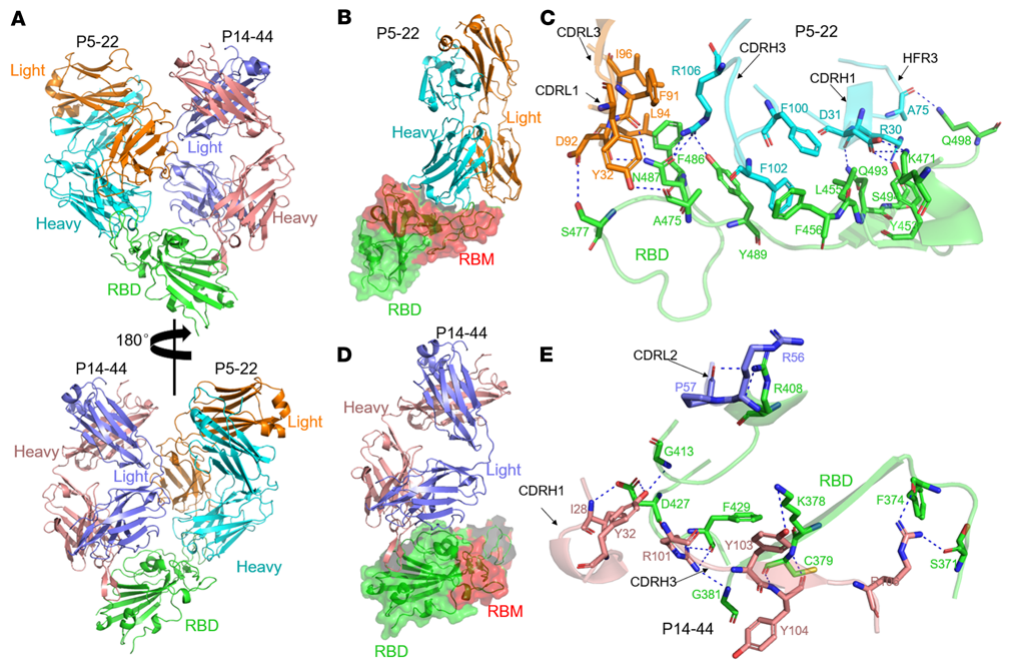
Overall structure and interactions of P14-44 Fab and P5-22 Fab with the spike RBD. The secondary structure elements of the spike RBD, heavy and light chains of P5-22 Fab, and heavy and light chains of P14-44 Fab are colored in green, cyan, orange, blue, and violet, respectively. The interacting residues are shown as sticks. The dashed lines represent hydrogen bonds and salt bridges. (**A**) Overall heterotrimeric structure of P5-22 Fab and P14-44 Fab in complex with the RBD. (**B**) Structure of P5-22 in complex with RBD. The surface of the RBD is colored in green and that of RBM in red. (**C**) Key interactions between P5-22 and the RBD. Two hydrophobic cores are formed between P5-22 and the RBD. (**D**) Structure of P14-44 in complex with the RBD. (**E**) Detailed interactions between P14-44 and the RBD.

Significantly, P5-22 recognizes an epitope that almost completely overlaps ACE2’s binding epitope in the receptor binding motif (RBM), suggesting that P5-22 and ACE2 would be sterically incompatible when synchronously binding to a single RBD (Supplemental Figure 11A). Five regions of P5-22 are involved in interacting with various regions of the RBD (Y449–F456, F486–Q498, K417, and A475) as follows: CDRH1, CDRH3, and HFR3 (framework region 3) of the heavy chain; and CDRL1 and CDRL3 of the light chain (Figure 5B). In addition to a salt bridge between D31 of CDRH1 and K417 of the RBD, various hydrogen bonds were found at the interface between P5-22 and the RBD (Figure 5C). It should be noted that residues L94 and I96 of CDRL3 and F486 of the RBD form a hydrophobic core that contributes to antigen binding. Furthermore, CDRH3 also interacts with the RBD via another hydrophobic interaction between F100 and F102 of CDRH3 and L455 and F456 of the RBD (Figure 5C).

In contrast to P5-22, P14-44 binds to a cryptic epitope on the RBD (distinct from the RBM), which is similar to that of COVA1-16 (Supplemental Figure 11, B and C, and ref. 36). Binding of P14-44 to the RBD is principally dominated by the heavy chain (Figure 5D), with the heavy chain contributing a buried surface area of 707.4 Å^2^ and the light chain contributing an area of only 159.5 Å^2^. Only CDRH1, CDRH2, and CDRL2 of P14-44 interact with the RBD (residues S371–G381, R408–G413, and P426–F429). Specifically, residues I28 and Y32 of CDRH1 form 3 hydrogen bonds with G413 and D427 of the RBD. Interestingly, in addition to forming a salt bridge (between Y103 of CDRH3 and K378 of RBD), CDRH3 interacts with the planar backbone conformation of the RBD via 8 hydrogen bonds. Additionally, CDRL2 of P14-44 contributes to the stabilization of antigen-antibody binding via 2 hydrogen bonds (Figure 5E). In total, 8 of 12 hydrogen bonds are formed between P14-44 and the RBD via the backbone atoms on the RBD side, suggesting that this antibody has unique features that enable it to overcome SARS-CoV-2 mutations. Based on the interaction between the key residues of P5-22 and P14-44, we constructed and expressed mutated RBDs (R408M, D427A, D427L, and F486R) to test antibody binding affinity. The results showed that D427A and D427L disrupted P14-44 binding to the RBD while F486R disrupted P5-22 binding to the RBD (Supplemental Figure 9), which confirmed that the observed crystal structures were physiologically relevant.

However, the D427 residue, which plays a critical role in stabilizing S trimers, is conserved across MERS-CoV, SARS-CoV-1, and SARS-CoV-2 (37), and may have a low mutation rate. D427 and D428 specifically interact with K986, which electrostatically stabilizes the spike in the closed state. The loss of such electrostatic contacts switches the S protein’s dynamic equilibrium by promoting a large allosteric change, and shifts the spike toward the open trimer state (37, 38), which is unfavorable for the viruses. Thus, P14-44 docking on functionally critical residues in the RBD provides the antibody a mechanism to prevent viral escape. Interestingly, because of the potential steric repulsions between CDRH3 and the side chains of the P384 mutations, P14-44 is also escaped by mutations at the P384R/D site but not by those at P384A (Figure 3C and Supplemental Figure 12).

**Figure 6:**
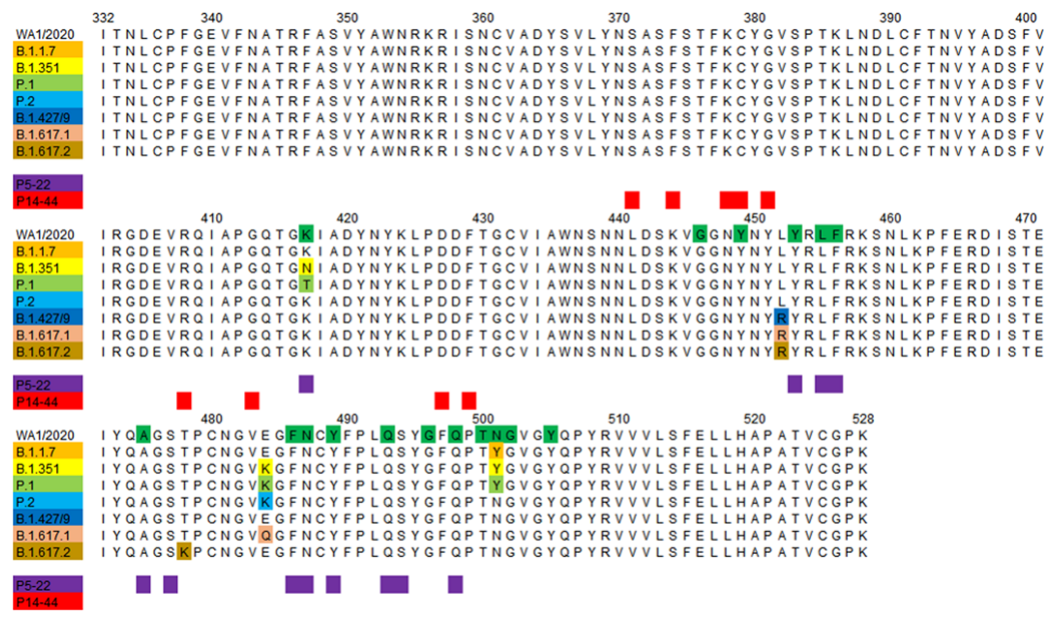
Multiple sequence alignment of spike RBD (aa 332–528) WA1/2020 and variants of concern (VOCs) of SARS-CoV-2 and the epitopes of P5-22 and P14-44 in the RBD. RBD residues involved in ACE2 binding are highlighted in green. Mutations within SARS-CoV-2 VOCs are highlighted in different colors. The epitope residues of P5-22 and P14-44 in the RBD are highlighted in purple and red, respectively.

To elucidate variability in the capacities of P5-22 and P14-44 to neutralize VOCs, a comparative analysis of the RBD of VOCs was performed. Almost all residues of P5-22 that interact with RBD (11 of 12) are shared by ACE2 (Figure 5C and Figure 6). Notably, only 1 epitope residue of P5-22 in the RBD (K417) showed a mutation in variant B.1.351 and P.1. However, the results of our measurement of the affinity between P5-22 and mutated RBDs indicated that certain residues had no significant impact on the binding between P5-22 and the RBD (Supplemental Figure 7). These results demonstrated that P5-22 can resist natural variants (Supplemental Figures 6 and 8). Sequence alignment indicated that P14-44 binds to an epitope that is entirely different from that of ACE2 as well as the mutated residues present in VOCs (Figure 5E and Figure 6). Significantly, epitope residues of P14-44 in the RBD in SARS-CoV-2, SARS-CoV-2 VOCs, SARS-CoV-1, Bat-CoV, RaTG13, and GD-Pangolin are all identical (Figure 6 and Supplemental Figure 13), raising the possibility that P14-44 may also block homologous coronaviruses. The RBD binding models of P14-44 and P5-22 indicated the potential benefits of developing IBI314 to counteract current and future SARS-CoV-2 VOCs at the clinical level.

### Polymeric P5-22 IgG but not bispecific IgG shows resistance to viral mutational escape.

**Figure 7:**
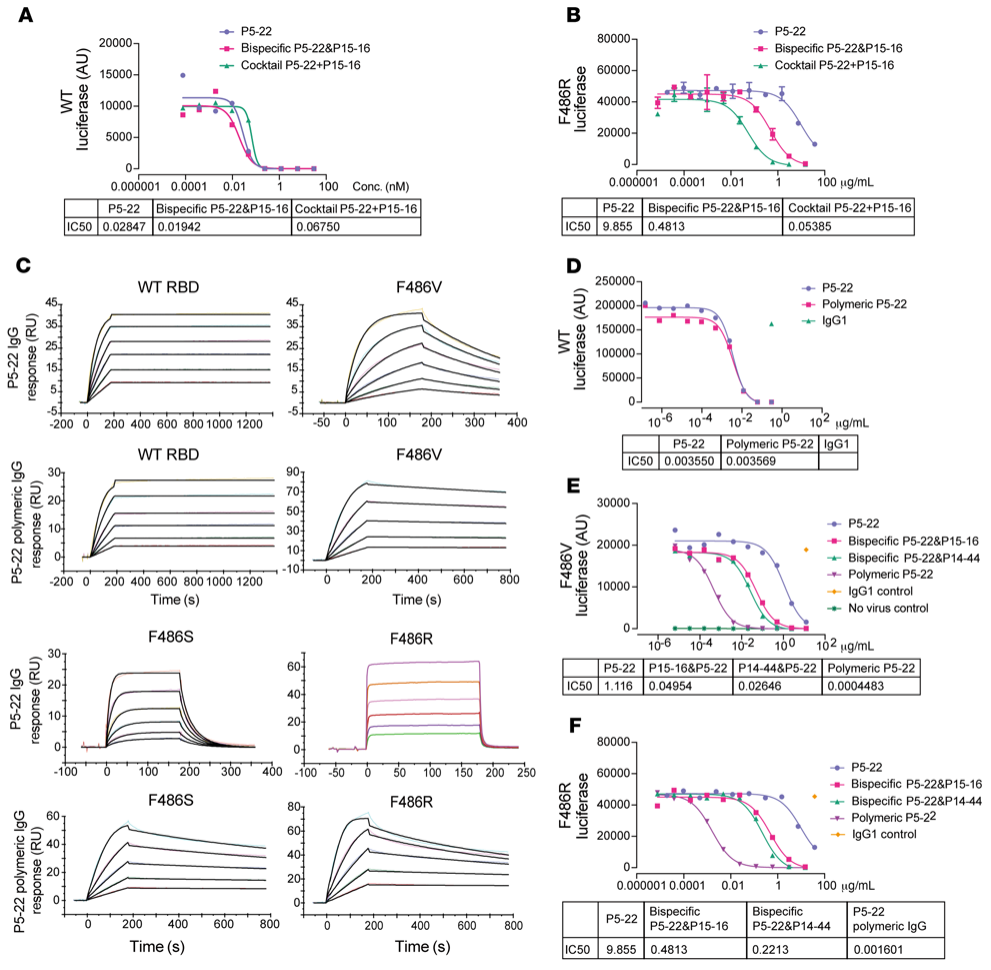
Polymeric IgGs but not bispecific IgGs potently neutralize constructed pseudovirus that can escape parental P5-22. (**A**) Neutralization curve of P5-22, bispecific P5-22 and P15-16, and cocktail of P15-16 and P5-22 for WT SARS-CoV-2 pseudovirus in vitro. (**B**) Neutralization curve of P5-22, bispecific P5-22 and P15-16, and cocktail of P15-16 and P5-22 for neutralization of F486R SARS-CoV-2 pseudovirus in vitro. (**C**) Binding of P5-22 or polymeric P5-22 to WT, F486V, F486S, and F486R RBDs. (**D**) Neutralization curves of P5-22 and polymeric P5-22 of WT SARS-CoV-2 pseudovirus in vitro. (**E** and **F**) Neutralization curve of P5-22, bispecific P5-22 and P15-16, bispecific P5-22 and P14-44, and polymeric P5-22 of F486V (**E**) or F486R (**F**) SARS-CoV-2 pseudovirus in vitro. Experiments were performed at least 3 times and 1 representative result is shown for each experiment.

We constructed a bispecific IgG antibody to test whether bispecific antibodies may overcome 1-arm escape as a strategy to prevent viral escape (39). Bispecific antibodies showed similar or even better potency than cocktails at neutralizing pseudoviruses generated using the WT spike protein (Figure 7A). However, the performance of bispecific antibodies in blocking F486R pseudovirus entry in vitro was worse than that of the cocktail (Figure 7B). This suggests that the avidity of neutralizing antibodies is critical for the neutralization of spike-mediated viral entry. We hypothesized that increasing neutralizing antibody valency may greatly enhance the avidity and result in a much more potent neutralization of the virus. Reportedly, polymeric IgG greatly enhances antibody valency and avidity, enabling more potent antibodies to be engineered (40). Since P5-22 is the antibody with the best potency and mutation resistance profiles, we constructed a polymeric P5-22 bearing 12 Fabs. The binding affinity of P5-22 measured by Biacore was 10^–12^ M (Figure 7C, Table 2, and Supplemental Figure 5A). F486 mutants, including F486V, F486S, and F486R, significantly decreased P5-22 binding (Figure 7C and Table 2). The polymeric form of P5-22, constructed by us, rescued P5-22 binding to all F486-mutant RBDs. Pseudovirus neutralization confirmed that the neutralization potency of P5-22 against F486V and F486R was fully rescued by polymeric IgG (Figure 7, D–F). Notably, the difference between the IC_50_ values of polymeric P5-22 and parental P5-22 IgG for neutralizing the F486R pseudovirus was over 6,000-fold (Figure 7F). Finally, we evaluated 2 benchmark nAbs developed in the clinic, REGN10933 and AZD8895, for their binding affinity for RBD F486R and F486I. Both have significantly decreased affinities for F486I and no detectable affinity for F486R. Polymeric IgG did not rescue parental IgG binding to F486R but completely rescued its affinity for F486I (Supplemental Figure 14, A and B). Polymeric IgGs also rescued the REGN10933 and AZD8895 parental IgG’s decreased potency in neutralizing F486I SARS-CoV-2 pseudovirus in vitro (Supplemental Figure 14C). Taken together, these results show that for polymeric IgG to overcome viral mutational escape of parental IgG, the parental IgG should have at least weak binding affinity for mutated RBDs.

## Discussion

Considering that SARS-CoV-2 has not been subjected to nAb selection pressure due to the limited clinical use of nAbs, it is imperative that screening for mutation-resistant nAbs that provide protection against current VOCs as well as future variants be developed. Here, we describe a reliable approach to map mutations that escape antibody binding to the RBD in a high-throughput manner. We developed a simple and rapid method to construct the yeast-display RBD-mutant libraries by using circular PCR and electroporation into yeast competent cells. In addition, by this method, we generated high-quality libraries in which 99% sequenced yeast clones are RBD proteins with exactly 1 amino acid substitution. It ensures the selected mutants that escape antibody binding are due to point mutation of the RBD without the complexity of combination mutations. In contrast, other reported libraries generated most RBD variants with multiple amino acid substitutions (29, 30). Thus, under this yeast library screening, in contrast to other reported nAbs, P5-22, P14-44, and P15-16 showed high resistance to viral mutations. Many broad-spectrum neutralizers have recently been reported. The common feature is that they bind a cryptic epitope away from the RBM. However, in vivo evolution of antibodies favors epitopes on the RBM (22). P5-22, which shows extremely high affinity for the spike protein (10^–12^ M *K_d_*) and is susceptible to only 1 hotspot, is one such anti-RBM antibody. Additionally, yeast library sequencing demonstrated that P15-16 has only 1 hotspot (K378) in the RBD, but to our surprise, the pseudovirus with mutant K378 was not infectious in vitro. This result indicated that SARS-CoV-2 cannot afford to mutate K378, implying a critical yet unrecognized role for K378 in viral fitness. Thus, P15-16 alone can be considered a mutation-proof single agent for COVID-19 treatment or prevention. We also discovered that several RBD-targeting antibodies occupying distinct bin 3–6 epitopes showed better neutralization activity than B38 (23) but not CB6 (Supplemental Figures 1 and 4). This provides further options to formulate a 3-antibody cocktail such as REGEN-COV (REGN10933 + REGN10987 + REGN10985; ref. 19) if needed. Finally, we found that polymeric IgG, rather than bispecific IgG, offers more potent neutralization against mutant viruses by enhancing avidity. Since antibody/antigen avidity is crucial for protection, bispecific antibodies are susceptible to viral escape via 1-arm binding. According to a recent report, using IgM increases valency and enhances potency via avidity (41). However, parental antibody selection is critical for multivalent antibodies to overcome resistance. IgM-06 still fails to neutralize SARS-CoV-2 K444R because IgG-06 cannot bind to RBD K444R (41). We observed similar failure of rescue of polymeric REGN-10933 and AZD8895 against the RBD F486R mutant due to complete loss of affinity of the parental IgG. By contrast, polymeric P5-22 rescued all F486-mutant RBD binding affinity and pseudovirus neutralization potency. This highlights the outstanding mutation-resistant property of P5-22; it still maintains weak binding affinity for RBD F486 mutants. We noticed that 2 other isolated human mutation-resistant antibodies, 2C08 (42) and S2E12 (20), target the F486 residue for RBD binding, similarly to P5-22. 2C08 and S2E12 have a high degree of sequence identity (95% amino acid identity) and are both encoded by IGHV1-58 and IGKV3-20. We found that P5-22 is different, in that it is encoded by IGHV3-11 and IGKV1-33, indicating its uniqueness in sequence as well as binding properties. 2C08 has hotspot residues G476, G485D, and F486 in the RBD, but P5-22 only has F486 in the RBD as a hotspot. This indicates that P5-22 can serve as an ideal parental antibody for multimeric engineering. Finally, given the fact that Eli Lilly and Regeneron’s cocktail fails to protect against the new omicron variant and none of omicron’s mutations hit our antibody’s escape hotspot, we speculate our P5-22/P14-44 cocktail would still neutralize the omicron variant and propose clinical development of these mutation-resistant nAbs to combat a possible serious threat of current and future variants.

## Methods

### PBMC processing.

PBMCs were maintained in heparinized blood collection tubes and shipped to Innovent Biologics from Tongji Hospital, Wuhan, China and The First Affiliated Hospital of Soochow University, Suzhou, China via cold-chain transportation within 24 hours of blood collection in hospitals. PBMCs were isolated via Ficoll gradient centrifugation (GE Healthcare) at 872*g* for 20 minutes with no brake on decelerating and stored in liquid nitrogen in the presence of FCS and DMSO.

### Single B cell sorting.

Human PBMCs were isolated via Ficoll density gradient centrifugation, and B cells were enriched using a B cell enrichment kit from Stemcell following the manufacturer’s protocol. Cells were then incubated with 200 μL APC-conjugated S protein trimer (Invitrogen, catalog A20186) and Alexa Fluor 488–conjugated S1 subunit (Invitrogen, catalog A20181) in combination with PE-Cy7 anti–human CD19 (BioLegend, catalog 302216) and APC-Cy7 anti–human CD20 (BioLegend, catalog 302314). Cells were stained in staining buffer (PBS plus 2% FBS) for 30 to 60 minutes on ice and then washed with 15 mL ice-cold staining buffer. Next, stained samples were sorted into cell lysis buffer in 96-well plates via a FACSAria II Cell Sorter (BD Biosciences). CD19^+^CD20^+^S trimer^+^S1^+^ live cells within the lymphocyte gate determined by forward scatter (FSC) and side scatter (SSC) were collected. The 96-well plates were then immediately frozen at –80°C for future use. 

### Single B cell VH and VL cloning and antibody expression.

Antibodies were identified and sequenced according to a previously described method (43). In brief, RNA from single cells was reverse transcribed (SuperScript IV Reverse Transcriptase, Thermo Fisher Scientific, 18090050) and cDNA was stored at –40°C or used for subsequent amplification of the variable IGH, IGL, and IGK genes via nested PCR, starting with 4 μL of cDNA as a template. All PCR reactions were performed in 96-well plates, in which each well contained a total of 20 μL consisting of 10 nM primer mix and 10 μL HotStart Plus (Qiagen). All nested PCR reactions using gene-specific primers or primer mixes were performed on 3.5 μL of unpurified first PCR product. Each round of PCR was performed for 50 cycles at 94°C for 30 seconds, 58°C for 30 seconds, and 72°C for 50 seconds. Amplicons from the first PCR reaction were used as templates for Sanger sequencing. Sequence analysis was performed using MEGA7 software (https://www.megasoftware.net). Sequences were then analyzed using IgBLAST (https://www.ncbi.nlm.nih.gov/igblast/) to identify germline V(D)J gene segments with the highest identity.

Cognate paired VH/VL regions were cloned into the expression vector pcDNA3.1 (Invitrogen, V79020). The light chain plasmid and the heavy chain plasmid of the same antibody were mixed at a 1:1 molar ratio and then transfected into HEK293F cells with polyethyleneimine. Next, 5 to 7 days later, when cell viability was less than 60%, the cell culture supernatant was collected and purified using a Protein A affinity column.

### Epitope binning assay.

Epitope binning of candidate antibodies was performed using a Fortebio Octet Red96e. Briefly, His-tagged SARS-CoV-2 S protein was loaded onto HIS1K sensors (Fortebio, 18–5120). Next, immobilized sensors were saturated with antibody 1, followed by antibody 2 for competition. Additional binding by the second antibody indicated a different binding epitope (noncompetitor), whereas absence of binding indicated the same binding epitope (competitor).

### Antibody/antigen affinity determination.

A Biacore T200 instrument was used to generate binding kinetics rates and affinity constants. First, the WT SARS-CoV-2 S RBD protein and the SARS-CoV-2 S RBD protein were individually immobilized on a CM5 sensor chip. Secondly, serially diluted antibodies were injected over the immobilized surface, allowing for a dissociation phase in the running buffer. The data were processed and fitted with Biacore T200 evaluation software, version 3.1, using a 1:1 binding model to determine the kinetics rates and affinity constants. Affinities of nAbs and RBD mutants were determined using the biolayer interferometry–based ForteBio Red96. The His-tagged RBD was loaded onto HIS1K biosensors (Fortebio, 18-5120) at 100 nM for 100 seconds. Afterwards, sensors were dipped into antibody solution at 100 nM for 100 seconds, and then dissociated in sample dilution buffer (1× PBS + 0.1% BSA + 0.05% Tween 20) for 120 seconds. Affinity data were assessed using ForteBio data analysis software version 10.0.

### Pseudoviruses.

The SARS-CoV-2 D614G (WT) B.1.1.7 (C726BGC050-1), B.1.351 (C8055GD080), and B.1.617.1 pseudoviruses were purchased from GenScript. The F486V pseudovirus was purchased from Vazyme (DD1411). Other SARS-CoV-2 pseudovirus variants with customized RBD mutations were constructed using Genewiz via customized orders.

### Pseudovirus neutralization assay.

HEK293 cells stably expressing ACE2 (HEK293/ACE2) were from GenScript (catalog M00770). HEK293/ACE2 cells were plated in a 96-well plate. After 8–10 hours, serially diluted antibodies ranging from 0.0001 to 100 nM were incubated with pseudovirus on ice for 1 hour. The mixture was added to the cells in a 96-well plate and cultured for 60 hours. A microplate reader was used to detect luciferase signal intensity.

### Mice, virus, and cells.

Specific pathogen–free 6-week-old BALB/c mice were purchased from Hunan SJA Laboratory Animal Co., Ltd. The mice were maintained at Guangzhou Medical University. The SARS-CoV-2 strains used in this study were isolated from COVID-19 patients in Guangzhou, China, including WT, alpha (B.1.1.7), beta (B.1.351), delta (B.1.617.2), and eta (B.1.525), passaged, and titered on Vero E6 cells. African green monkey kidney–derived Vero E6 cells were grown in Dulbecco’s modified Eagle’s medium (DMEM; Gibco) supplemented with 10% FBS. The human serotype 5 adenoviral vector (Ad5) expressing human ACE2 regulated by the CMV promoter has been previously described (24, 44, 45). All work with SARS-CoV-2 was conducted at the Guangzhou Customs District Technology Center Biosafety Level 3 (BSL-3) Laboratory.

### Viral titer determination.

Focus-forming assays (FFAs) were performed to determine viral titers. Lungs were removed in PBS and homogenized using a manual homogenizer. The virus was titered on Vero E6 cells. Vero E6 cells were seeded on 96-well plates overnight and grown into confluent monolayers. Fifty microliters of 10-fold-diluted SARS-CoV-2 stock or supernatant of lung homogenate was added to each well of a 96-well plate and adsorbed at 37°C for 1 hour with rocking every 10 minutes. The virus or the supernatant of the lung homogenate was removed and covered with 100 μL MEM containing 1.2% carboxymethylcellulose. The overlay was discarded 24 hours after infection, and the cell monolayer was fixed with 4% paraformaldehyde solution for 2 hours at room temperature. Following permeabilization with 0.2% Triton X-100 for 20 minutes at room temperature, the plates were sequentially stained with cross-reactive rabbit anti–SARS-CoV-2 N IgG (Sino Biological Inc., catalog 40143-T62) as the primary antibody and HRP-conjugated goat anti–rabbit IgG (H+L) (Jackson ImmunoResearch, 109-035-088) as the secondary antibody at 37°C for 1 hour. The reactions were developed using KPL TrueBlue peroxidase substrate. The number of SARS-CoV-2 foci was calculated using a CTL ImmunoSpot S6 Ultra Reader (Cellular Technology Ltd). Viral titers were calculated as focus-forming units (FFU) per gram of tissue.

### SARS-CoV-2 exposure studies in Ad5-hACE2–transduced mice.

Ad5-hACE2–transduced BALB/c mice were infected with SARS-CoV-2/human/CHN/IQTC01/2020 strain (accession number MT123290) of SARS-CoV-2 as described previously (24). Mice were lightly anesthetized with isoflurane and transduced intranasally with 2.5 × 10^8^ PFU of Ad5-hACE2 in 75 μL of DMEM. Five days after transduction, mice were infected intranasally (i.n.) with SARS-CoV-2 (1 × 10^5^ PFU) in a total volume of 50 μL of DMEM. BALB/c mice in the therapeutic group (*n =* 11) were injected intraperitoneally (i. p.) with 2, 10, or 50 mg/kg of IBI314 cocktail or negative control hIgG (50 mg/kg) 1 day following infection. To obtain SARS-CoV-2 titers, lungs (*n =* 3 mice) were removed in PBS at 3 dpi and homogenized. Viral titers of clarified supernatants from Vero E6 cells were assayed and expressed as FFU per gram of tissue. Body weight (*n =* 5 mice) was monitored daily throughout the postinfection follow-up period. Animals were anesthetized and transcranially perfused with PBS, followed by zinc formalin. The lungs were removed, fixed in zinc formalin, and embedded in paraffin. Sections were stained with hematoxylin and eosin (*n =* 3 mice). Edema, necrotic cellular debris, and mononuclear cells were scored according to the following criteria: 0, none; 1, uncommon detection in <5% lung fields; 2, detectable in up to 33% of lung fields; 3, detectable in up to 33%–66% of lung fields; and 4, detectable in >66% of lung fields. Neutrophil infiltration was scored according to the following criteria: 0, within normal limits; 1, scattered polymorphonuclear cells (PMNs) sequestered in septa; 2, criterion 1 plus solitary PMNs extravasate in airspaces; and 3, criterion 2 plus small aggregates in the vessel and airspaces. Lung homogenates were also used for total RNA extraction using TRIzol reagent (Invitrogen, 15596026) to identify changes in the RBD associated with IBI314 cocktail escape, and viral RNA was extracted for Sanger sequencing using specific primers to determine the mutation sites. Sequences were analyzed using BioEdit software (https://bioedit.software.informer.com/7.2/). All work with SARS-CoV-2 was conducted at the Guangzhou Customs District Technology Center BSL-3 Laboratory.

### Crystallization and structure determination.

To obtain the RBD (aa 332–528) complexed with Fab P14-44, RBD_332–528_ was mixed with Fab P14-44 at a molar ratio of 1.25:1 and incubated overnight at 4°C. The RBD_321–591_–Fab P14-44–Fab P5-22 complex was formed by mixing individual components in a 1.25:1:1 molar ratio and incubating overnight at 4°C. Subsequently, these protein complexes were purified via gel filtration using a Superdex 16/200 column (GE Healthcare) and concentrated to approximately 20 mg/mL in 150 mM NaCl and 20 mM HEPES pH 7.0 for crystallization screening.

The splitting-drop vapor diffusion method was applied to obtain RBD_332–528_–Fab P14-44 crystals at 291 K by mixing 0.2 μL of protein complexes with an equal volume of reservoir solution. Optimized crystals were achieved in 100 mM sodium citrate (pH 5.6), 20% (w/v) PEG 4000, and 20% (v/v) isopropanol.

The hanging-drop vapor diffusion method was used to acquire crystals of the RBD_321–591_–Fab P14-44–Fab P5-22 complex at 291 K by mixing 1 μL of protein complexes with 1 μL of reservoir solution. Diffraction-quality crystals of the RBD_321–591_–Fab P14-44–Fab P5-22 complex were grown in 1% (w/v) tryptone, 20% (w/v) PEG 3350, and 50 mM HEPES pH 7.0.

For data collection, single crystals were flash-cooled in liquid nitrogen after immersion in a cryoprotectant composed of 15% (v/v) glycerol in the reservoir solution for a few seconds. All diffraction data were collected at the beamline BL19U1 at the Shanghai Synchrotron Radiation Facility (SSRF) at a wavelength of 0.97853 Å at 100 K. All collected data were processed using the HKL2000 software package (46). Initial phases were solved via the molecule replacement method by Molrep from the CCP4i software package (47, 48), using SARS-CoV-2 RBD/COVA1-16 Fab (PDB ID: 7JMW) and SARS-CoV-2 RBD/REGN10933 Fab (PDB ID: 6XDG) as search models (3, 36). Subsequent model building and refinement were achieved using COOT and Refmac 5, respectively (49, 50). All structural figures were prepared using PyMOL software (51). Data collection and model refinement data are presented in Table 1.

### Yeast RBD-mutant library construction.

The DNA fragment encoding the spike RBD of SARS-CoV-2 was synthesized and cloned into the backbone vector pYDC011 (a yeast surface-display expression vector) by Genewiz. Mutagenesis of the SARS-CoV-2 RBD was performed using the circular PCR method. Briefly, we designed a total of 201 mutagenic oligonucleotides with degenerate NNK codons to introduce exactly 1 amino acid mutation per variant. Each oligonucleotide containing 1 NNK codon diversifies a specific residue of the RBD protein into all possible amino acids. A total of 17 separate libraries were constructed to cover all amino acids in the RBD protein. Each library contained 384 DNA unique members, except the last library which had a diversity of 288 DNA members. Thus, in aggregate the libraries had a diversity of approximately 6500 point-mutated RBDs. For each library, the PCR reaction included approximately 12 mutagenic oligonucleotides as the forward primer, 1 adjacent oligo as the reverse primer, pYDC364 plasmid containing the WT RBD gene as the template, and Primerstar polymerase (Takara, R045A). The reaction was started at 95°C for 5 minutes, followed by 20 cycles of 95°C for 30 seconds, 60°C for 30 seconds, 72°C for 5 minutes, and finally 72°C for 10 minutes. The PCR products were gel purified and treated with the enzyme DpnI (Thermo Fisher Scientific, FD1703) to digest the plasmid template. The cleaned PCR products were electrotransformed into the EBY stain of *Saccharomyces cerevisiae* (ATCC, MYA4941) and cultured overnight in rich YPD medium (1% yeast extract, 2% peptone, and 2% glucose) with shaking at 30°C. Recombinant strains were grown in 50 mL selective SD-Trp medium (Clontech, ST0041) and expression of RBDs was induced in YPGP medium (1% yeast extract, 2% peptone, 2% galactose, 1.36% Na_2_HPO_4_·12H_2_O, 0.978% NaH_2_PO_4_·2H_2_O) at 20°C with shaking at 225 rpm for 24 hours. Colony-forming unit (CFU) counts from plates of serial dilutions indicated a transformation efficiency greater than 1 × 10^5^. At least 100 clones were randomly selected and cultured in 1 mL liquid SD-Trp media at 30°C and 250 rpm overnight for sequencing to verify library construction. After verification, 17 libraries were equally mixed and subjected to immunofluorescence labeling. Transformed yeast libraries were aliquoted at 1× 10^8^ CFU each and stored at –80°C.

### FACS of yeast libraries.

Yeast library stocks were diluted in 250 mL YP medium with 2% galactose and grown for 24 hours at 25°C in a shaking incubator to induce RBD surface expression. Approximately 1 × 10^7^ induced cells were collected, washed twice with PBS plus BSA (0.1 mg/mL), and diluted in 1 mL PBS-BSA. Biotinylated ACE2 protein at 150 nM (Acro, AC2-H82E6) and anti-RBD antibody at EC_50_ binding concentration (approximately 1–3 nM) were added to the cells and incubated for 1 hour at room temperature. Cells were washed twice and stained with anti–human Fc-FITC (Sigma-Aldrich, F9512) and streptavidin-PE (eBioscience, 12-4317-87) secondary antibodies. Labeled cells were washed twice with PBS-BSA and suspended in 1 mL PBS. An escapee gate was drawn to capture the ACE2-binding but not non–antibody-binding yeast clones. Yeast libraries were sorted using a BD FACSAria II. Cells were sorted into 5 mL FACS tubes containing 5 mL of SD-Trp medium and later cultured at 30°C and 225 rpm.

### Plasmid extraction and RBD sequencing analysis.

Plasmid samples were prepared from overnight cultures of 20 OD units of preselected or antibody-escaped yeast populations (Takara Yeast Plasmid Miniprep, 630467). Plasmids were electrotransformed into TG1 cells (from Lucigen) and cultured on 2XYT-ampicillin agar plates (1.6% tryptone, 1% yeast extract, 0.5% sodium chloride, 1.5% agar, and 100 μg/mL ampicillin) at 37°C for 12–18 hours. Resistant clones were randomly selected for Sanger sequencing using promoter-specific primers. The candidate antibody escape samples from the P5-22, P5-16, or P14-44 clones had at least 5 times as many sequencing counts as FACS-selected cells. A reference antibody escape sample usually has more than 300 sequencing counts. Raw sequencing data were aligned and analyzed using MEGA7 software. Mutation-level escape scores and site-level measurements were calculated according to a previous study (29). Briefly, site-level escape was estimated as the sum of all mutation-level escape fractions at a site. For each antibody, sites with strong escape mutations were defined as having at least 3% site-level escape ratios and were chosen to be displayed in the logo plots.

### Mutant escape profiling.

The static logo plots were created by dmslogo (https://jbloomlab.github.io/dmslogo/) version 0.6.2; the color of the mutations indicates the expression of RBDs derived from published data (52). Sarbecovirus variation of each mutation position was calculation by Sarbecovirus RBD region, which was downloaded from GitHub (https://github.com/jbloomlab/SARSr-CoV_RBD_MAP/blob/main/data/RBD_aa_aligned.fasta). Colors from light blue to black represent an increase in the mutation frequency. SARS-CoV-2 variations were calculated using spike sequences from the GISAID database (53). All spike sequences were aligned using MAFFT version 7 (54) with Wuhan-Hu-1 as the reference sequence. In the RBD region, sequences with gaps, ambiguous amino acids, or more than 8 amino acid differences from the Wuhan-Hu-1 sequence were removed, leaving 3,722,275 sequences for calculating variations.

### Construction of polymeric P5-22.

In order to generate polymeric P5-22, a human IgM heavy chain tailpiece was fused onto the IgG1 heavy chain C-terminus of the P5-22 antibody (EU numbering). In addition, heavy chain residue L309 in the P5-22 antibody was converted to the corresponding cysteine residue in IgM (40, 55).

### Statistics.

Statistical analyses were performed using GraphPad Prism software, version 8.00. The Student’s *t* test was used to analyze differences between mean values of groups. All results are expressed as the mean ± standard error of the mean (SEM). Statistical significance was set at a *P* value of 0.05 or less (*P ≤ 0.05; **P ≤ 0.01; ***P ≤ 0.001; ****P ≤ 0.0001).

### Data presentation.

Figures were arranged in Adobe Illustrator 2020. Statistical analyses were performed using GraphPad Prism 8.0 software. FACS data were analyzed and plotted by FlowJo X (Tree Star).

### Study approval.

Written informed consent was obtained from the patients and the study was approved by the ethics review boards at Tongji Hospital (Wuhan, China), The First Affiliated Hospital of Soochow University (Suzhou, China), and also The Third People’s Hospital of Changzhou (Changzhou, China). All animal protocols were approved by the Institutional Animal Care and Use Committee of Guangzhou Medical University.

## Author contributions

J Zou conceived the experiments and wrote the manuscript with input from all coauthors. L Li led antibody discovery. SZ led protein expression and purification. PZ and TJ solved the crystal structure of the IBI314-RBD complex. SH, XL, and PZ designed the yeast RBD library and performed the data analysis. WL, Y Wang, and J Zhao coordinated and evaluated the in vitro and in vivo authentic-virus experiments. DY designed the polymeric IgG. L Liu, DW, MX, FZ, MZ, ZW, XG, MN, XL, Y Wu, MH, J Li, XC, and ZK performed experiments. NZ, MY, CG, J Liu, HJ, and JH provided funding and critical support for the work. J Zhou, JH, and TX provided patient samples.

## Supplementary Material

Supplemental data

Supplemental table 1

Supplemental table 2

Supplemental table 3

## Figures and Tables

**Table 1:**
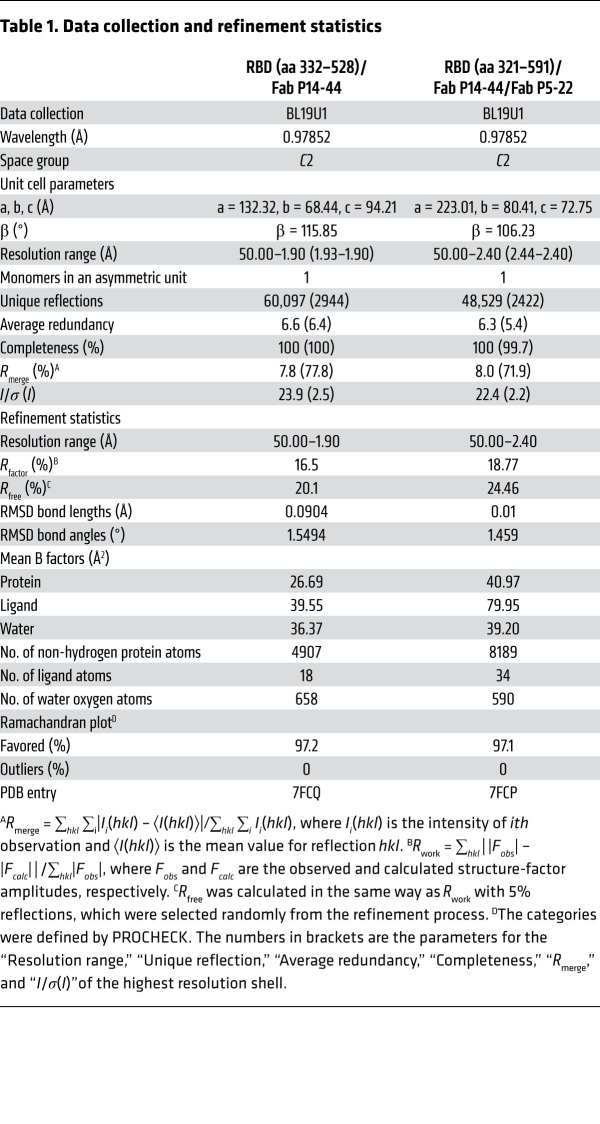
Data collection and refinement statistics

**Table 2:**
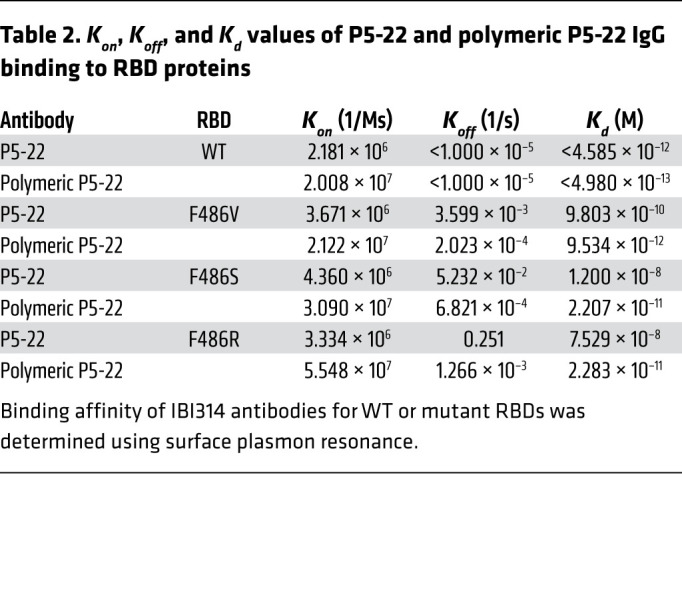
*K_on_*, *K_off_*, and *K_d_* values of P5-22 and polymeric P5-22 IgG binding to RBD proteins

## Version 1 02/15/2022

Electronic publication

## Version 2 02/02/2022

In-Press Preview
