# Supplementary material for: Ultrapotent neutralizing antibodies against SARS-CoV-2 with a high degree of mutation resistance
Source: J Clin Invest. 2022 Feb 15;132(4):e154987. doi: 10.1172/JCI154987 (PMC8843702; doi:10.1172/JCI154987)
Supplement: Supplemental data [file jci-132-154987-s012.pdf]

Supplemental Material

Supplemental Figure 1 *In vitro* screening of potential blockers for pseudovirus entry.

Figure S1

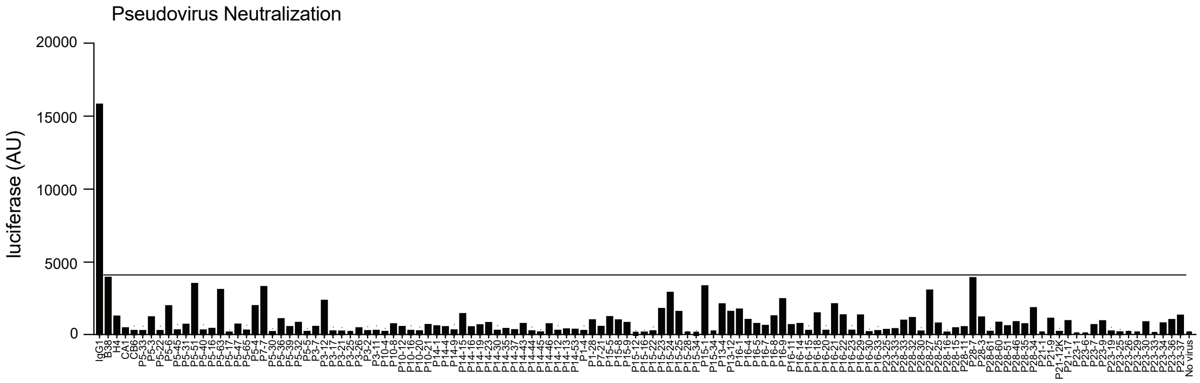

The 293-hACE2 cells were infected with a pseudovirus carrying the luciferase gene. Neutralizing antibodies, including benchmarks B38, H4, CA1, and CB6, were included for reference. Illustrated antibodies show equal or higher potency than B38 (black line) at 100 nM for pseudovirus neutralization at 72 h.

Supplemental Figure 2 Epitope binning information of 49 lead candidates indicate 6 distinct bins.

Figure S2

A

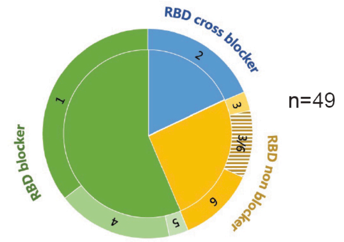

B

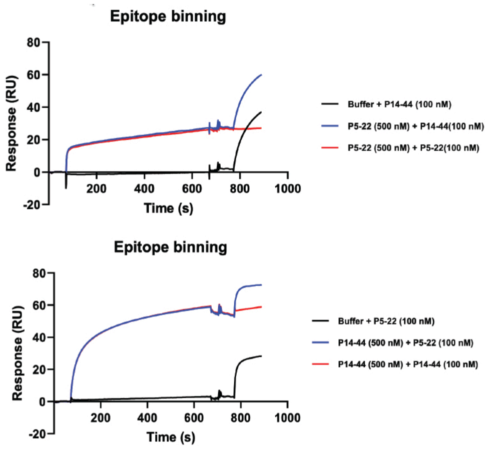

C

Bin	Reference antibody
1	P5-22
2	P15-16
3	P10-16
4	P5-65
5	P5-30
6	P5-39

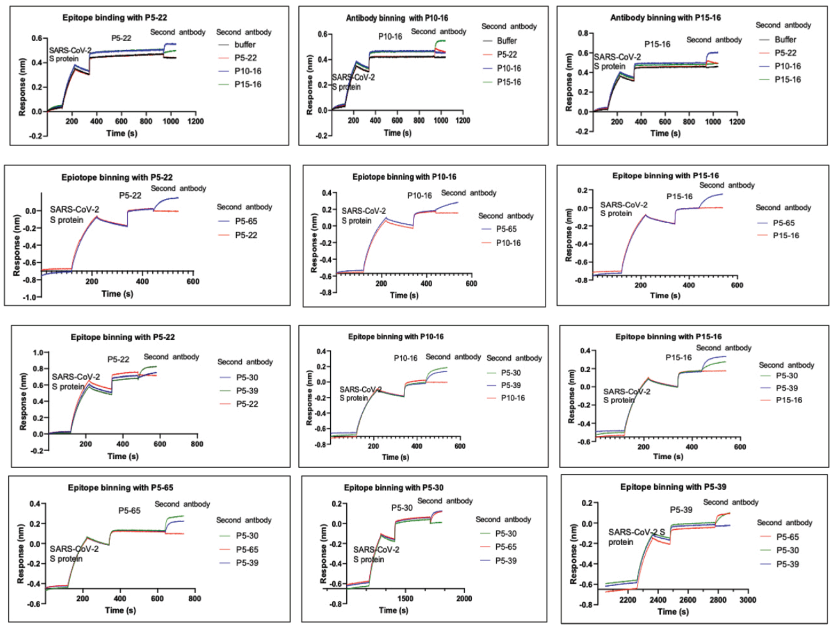

(A) Pie chart summary of 49 candidate antibody epitope binning results. (B) Epitope binning of P5-22 and P14-44. P14-44 recognized a distinct epitope from P5-22 in binding with SARS-CoV-2 S protein (C) Epitope binning experiment using reference antibody pairing showed 6 distinct bins of RBD binders.

Supplemental Figure 3 Pseudovirus neutralization curve of selected antibodies

Figure S3

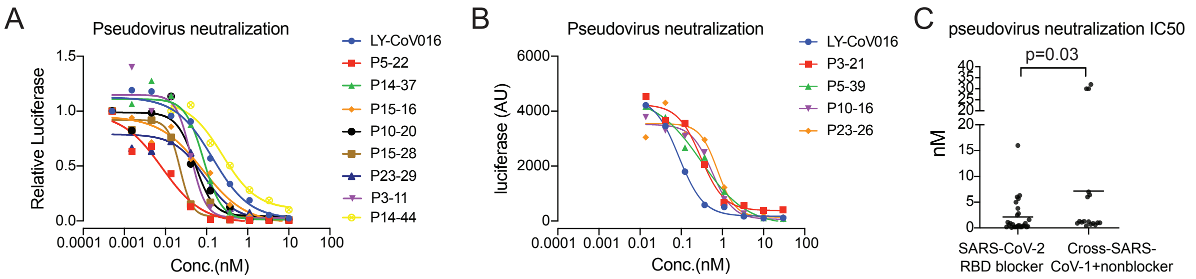

(A, B) Representative neutralization curve of selected RBD blockers (A) and non-blockers in the pseudovirus neutralization assay (B) compared to the benchmark antibody LY-CoV016 (CB6). (C) Comparison of the IC_{50} values of RBD blockers and non-blockers.

Supplemental Figure 4. Binding affinity of IBI314 antibodies with S proteins by SPR

Figure S4

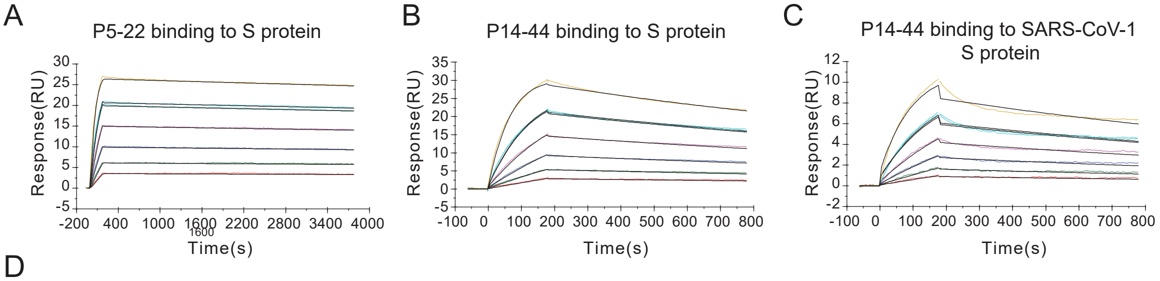

D

Antigen	Analyte	Run NO.	Ka(1/Ms)	Kd(1/s)	KD (M)	Mean KD (M) ± SD
SARS-CoV-2 S protein	P5-22	1	3.30E+06	<1.00E-5	<3.03E-12	<3.34E-12±2.91E-13
		2	2.97E+06	<1.00E-5	<3.37E-12	
		3	2.77E+06	<1.00E-5	<3.61E-12	
SARS-CoV-2 S protein	P14-44	1	2.67E+05	8.17E-05	3.07E-10	5.54E-10±2.17E-10
		2	2.45E+05	1.76E-04	7.16E-10	
		3	2.25E+05	1.44E-04	6.38E-10	
SARS-CoV-1 S protein	P5-22	1	No binding			
		2	No binding			
		3	No binding			
SARS-CoV-1 S protein	P14-44	1	1.59E+05	5.77E-04	3.62E-09	4.29E-9±8.60E-10
		2	1.53E+05	8.05E-04	5.26E-09	
		3	1.52E+05	6.06E-04	3.99E-09	

Binding affinity of IBI314 antibodies were determined using surface plasmon resonance (SPR). Binding of P5-22 (A) and P14-44 (B) to the spike (S) protein of SARS-CoV-2 and (C) binding of P14-44 to S protein of SARS-CoV-1 (D) Kinetics and affinity values of IBI314 antibodies were obtained in triplicate (C).

Supplemental Figure 5: IBI314 cocktail treatment did not induce escape mutants of SARS-CoV-2 in mice.

Figure S5

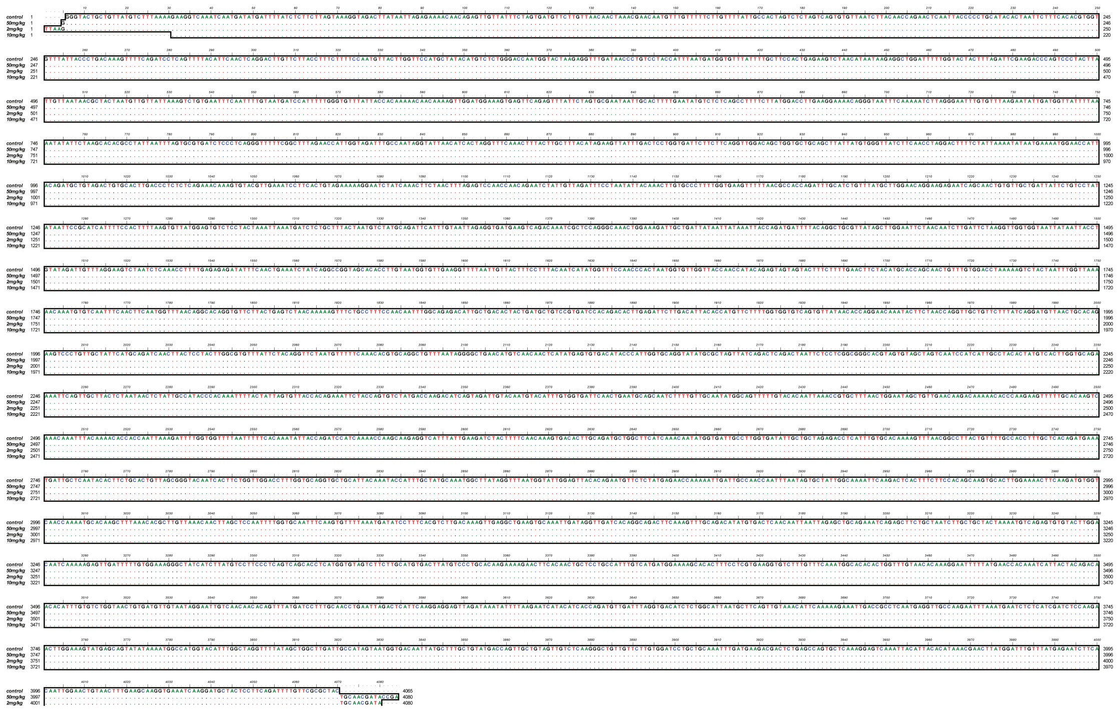

Viral spike protein sequences were obtained from the lungs of 3dpi Ad5-hACE2 mice and the original virus strain. Sanger sequencing was performed using specific primers to determine mutant sites. Sequences were analyzed using BioEdit software.

Supplemental Figure 6 Brief map of mutations that escape binding by the Eli Lilly cocktail antibodies

Figure S6

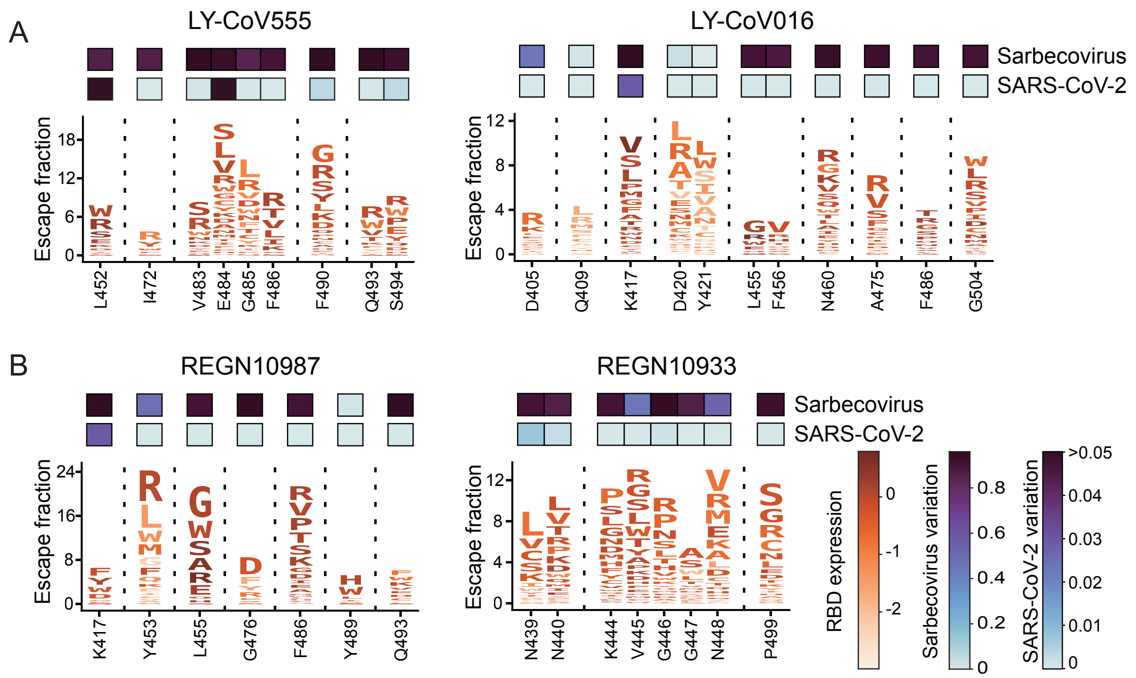

and REGN-CoV2 antibodies.

Logo plots of the map of mutations that escape binding of Eli Lilly cocktail antibodies (A) and REGN-CoV2 antibodies (B) The height of each letter is proportional to how strongly that amino-acid mutation mediates escape. Reported mutations among SARS-CoV-2 sequences in GISAID and RBD sequence alignment among Sarbecoviruses are used to generate heat maps to visualize mutations on each residue reflecting the likelihood of mutation occurance.

Supplemental Figure 7 Affinity kinetics of P5-22, P14-44, and P15-16 with reported mutated RBD from different reported SARS-CoV-2 virus variants by biolayer interferometry (BLI).

Figure S7

Sample ID	KD (M) antigens on sensor tip, P15-16 in solution 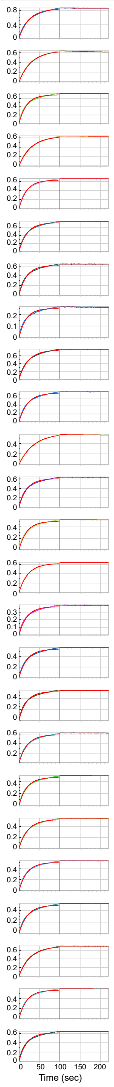	Response	KD (M)	kon(1/Ms)	kdis(1/s)	KD (M) antigens on sensor tip, P5-22 in solution 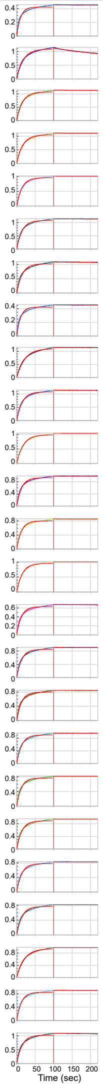	Response	KD (M)	kon(1/Ms)	kdis(1/s)	KD (M) antigens on sensor tip, P14-44 in solution 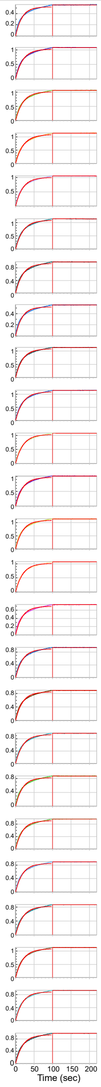	Response	KD (M)	kon(1/Ms)	kdis(1/s)
WT		0.8492	4.95E-10	4.04E+05	2.00E-04		1.2601	3.29E-10	6.09E+05	2.00E-04		1.2043	4.93E-10	4.06E+05	2.00E-04
F486V		0.7087	4.05E-10	4.94E+05	2.00E-04		1.1329	3.15E-09	5.34E+05	1.68E-03		1.0574	3.89E-10	5.14E+05	2.00E-04
E406W		0.7111	3.70E-10	5.40E+05	2.00E-04		1.1257	3.12E-10	6.41E+05	2.00E-04		1.0511	3.98E-10	5.02E+05	2.00E-04
K417V		0.6224	5.96E-10	3.36E+05	2.00E-04		1.01	3.36E-10	5.95E+05	2.00E-04		1.1233	4.22E-10	4.74E+05	2.00E-04
N481L		0.6537	3.80E-10	5.26E+05	2.00E-04		1.096	2.86E-10	6.99E+05	2.00E-04		1.0361	3.89E-10	5.14E+05	2.00E-04
S477A		0.7546	4.01E-10	4.98E+05	2.00E-04		1.1368	2.95E-10	6.79E+05	2.00E-04		1.1252	4.04E-10	4.96E+05	2.00E-04
N450L		0.6553	3.80E-10	5.27E+05	2.00E-04		0.9864	2.66E-10	7.53E+05	2.00E-04		0.9988	3.81E-10	5.25E+05	2.00E-04
Y508H		0.2636	3.46E-10	5.79E+05	2.00E-04		0.4523	2.08E-10	9.61E+05	2.00E-04		0.547	3.48E-10	5.76E+05	2.00E-04
F456V		0.7589	4.49E-10	4.45E+05	2.00E-04		1.1317	4.32E-10	4.63E+05	2.00E-04		1.1222	3.80E-10	5.27E+05	2.00E-04
L455V		0.7468	4.16E-10	4.80E+05	2.00E-04		1.1375	2.99E-10	6.68E+05	2.00E-04		1.1244	4.00E-10	5.00E+05	2.00E-04
K417N-E484K -N501Y		0.5587	7.23E-10	2.77E+05	2.00E-04		1.0372	3.55E-10	5.64E+05	2.00E-04		1.073	4.05E-10	4.94E+05	2.00E-04
K444Q		0.63	3.85E-10	5.20E+05	2.00E-04		0.9802	2.61E-10	7.66E+05	2.00E-04		1.0068	3.81E-10	5.25E+05	2.00E-04
V445A		0.5188	3.85E-10	5.19E+05	2.00E-04		0.8493	2.56E-10	7.81E+05	2.00E-04		0.9025	3.76E-10	5.32E+05	2.00E-04
L452R		0.6358	4.46E-10	4.48E+05	2.00E-04		1.012	3.04E-10	6.58E+05	2.00E-04		1.0501	3.96E-10	5.05E+05	2.00E-04
Y453F		0.3865	3.80E-10	5.26E+05	2.00E-04		0.6638	2.52E-10	7.95E+05	2.00E-04		0.7238	3.62E-10	5.52E+05	2.00E-04
V483A		0.561	3.95E-10	5.07E+05	2.00E-04		0.8982	2.68E-10	7.47E+05	2.00E-04		0.937	3.85E-10	5.19E+05	2.00E-04
F490L		0.5427	3.59E-10	5.57E+05	2.00E-04		0.8392	2.56E-10	7.81E+05	2.00E-04		0.8816	3.72E-10	5.38E+05	2.00E-04
N439K		0.6049	4.07E-10	4.91E+05	2.00E-04		0.9544	2.72E-10	7.35E+05	2.00E-04		0.9695	3.93E-10	5.09E+05	2.00E-04
N450D		0.4867	3.82E-10	5.23E+05	2.00E-04		0.8114	2.42E-10	8.28E+05	2.00E-04		0.863	3.70E-10	5.41E+05	2.00E-04
L455F		0.5411	4.45E-10	4.49E+05	2.00E-04		0.9402	2.80E-10	7.15E+05	2.00E-04		0.9497	3.73E-10	5.36E+05	2.00E-04
A475V		0.5189	3.88E-10	5.15E+05	2.00E-04		0.8235	2.69E-10	7.43E+05	2.00E-04		0.8848	3.78E-10	5.29E+05	2.00E-04
E484Q		0.5123	4.13E-10	4.84E+05	2.00E-04		0.8163	2.82E-10	7.09E+05	2.00E-04		0.8572	3.99E-10	5.01E+05	2.00E-04
G485D		0.6821	5.49E-10	3.65E+05	2.00E-04		0.9659	4.10E-10	4.88E+05	2.00E-04		1.1202	4.00E-10	5.00E+05	2.00E-04
F490P		0.5793	3.77E-10	5.31E+05	2.00E-04		0.8954	2.55E-10	7.83E+05	2.00E-04		0.9237	3.77E-10	5.30E+05	2.00E-04
Q493K		0.6344	3.73E-10	5.37E+05	2.00E-04		1.0857	2.94E-10	6.81E+05	2.00E-04		0.9768	3.87E-10	5.17E+05	2.00E-04

Affinities of nAbs (100 nM) and RBD mutants (100 nM) were determined by biolayer interferometry (BLI) using ForteBio Red96e. Affinity data were analyzed using the ForteBio data analysis software version 10.0.

Supplemental Figure 8 Neutralization potency of prevailing pseudovirus VOCs by IBI314 and its components.

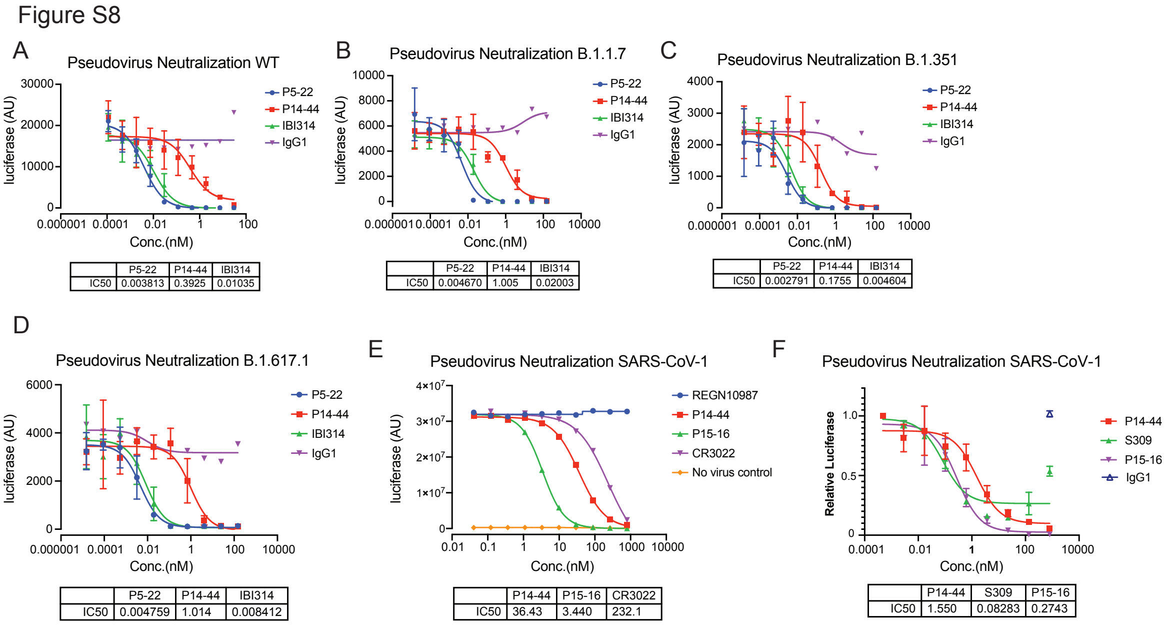

A

	P5-22	P14-44	IBI314
IC50	0.003813	0.3925	0.01035

B

	P5-22	P14-44	IBI314
IC50	0.004670	1.005	0.02003

C

	P5-22	P14-44	IBI314
IC50	0.002791	0.1755	0.004604

D

	P5-22	P14-44	IBI314
IC50	0.004759	1.014	0.008412

E

	P14-44	P15-16	CR3022
IC50	36.43	3.440	232.1

F

	P14-44	S309	P15-16
IC50	1.550	0.08283	0.2743

(A, B, C, D) Representative neutralization curve of IBI314 and its mAb components in neutralizing WT (A), B.1.1.7 (B), B.1.351 (C), and B.1.617.1 (D) pseudovirus. (E) Representative neutralization curves of P14-44 and P15-16 in SARS-CoV-1 pseudovirus neutralization assay, compared to REGN10987 and CR3022.

Supplemental Figure 9: Affinity kinetics of P5-22, P14-44 and P15-16 with indicated mutated RBDs by BLI.

Figure S9

Sample ID	KD (M) antigens on sensor tip, P15-16 in solution	Response	KD (M)	kon(1/Ms)	kdis(1/s)	KD (M) antigens on sensor tip, P5-22 in solution	Response	KD (M)	kon(1/Ms)	kdis(1/s)	KD (M) antigens on sensor tip, P14-44 in solution	Response	KD (M)	kon(1/Ms)	kdis(1/s)
D427A	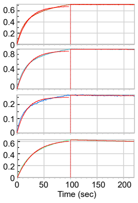	0.7007	4.11E-10	4.86E+05	2.00E-04	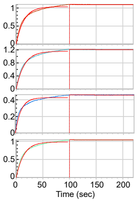	1.0894	2.86E-10	7.00E+05	2.00E-04	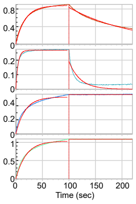	0.8954	1.61E-08	4.98E+05	8.03E-03
D427L		0.7925	4.16E-10	4.81E+05	2.00E-04		1.207	3.00E-10	6.66E+05	2.00E-04		0.2733	2.57E-08	1.87E+06	4.80E-02
F486R		0.7725	4.31E-10	4.64E+05	2.00E-04		0.5083	2.29E-08	1.01E+06	2.30E-02		1.1267	4.11E-10	4.87E+05	2.00E-04
R408M		0.6184	1.05E-09	3.30E+05	3.46E-04		1.0281	2.82E-10	7.09E+05	2.00E-04		1.0701	3.85E-10	5.19E+05	2.00E-04

Affinities of nAbs (100 nM) and RBD mutants (100 nM) were determined using the biolayer interferometry-based ForteBio Red96e. Affinity data were analyzed using the ForteBio data analysis software version 10.0.

Supplemental Figure 10 Comparison of P14-44 Fab from binary complex and ternary complex.

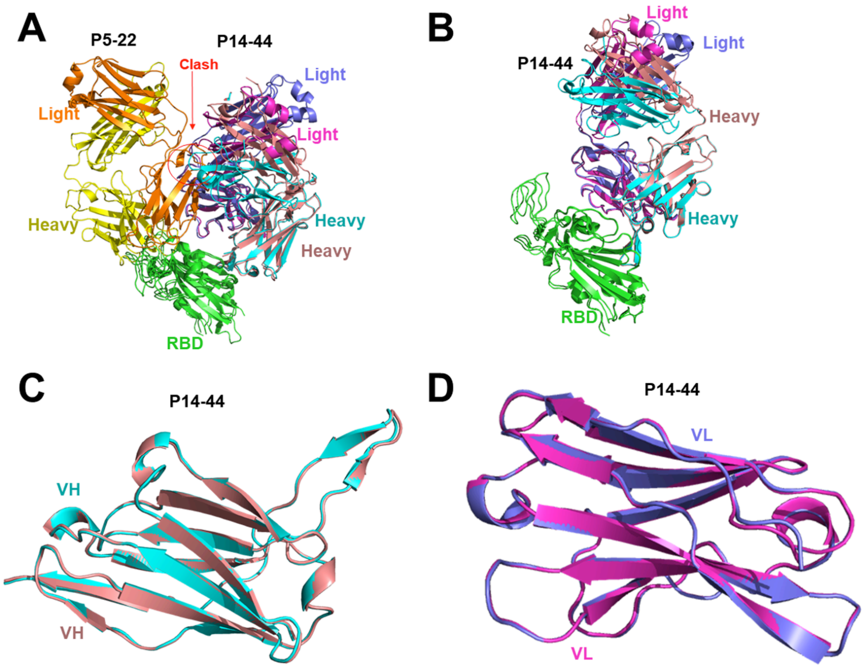

A
P5-22
P14-44
Clash
Light
Light
Light
Heavy
Heavy
Heavy
RBD
B
Light
Light
P14-44
Heavy
Heavy
RBD
C
P14-44
VH
VH
D
P14-44
VL
VL

Residues in the heavy and light chains of P14-44 Fab in the binary complex and ternary complex are colored in cyan, magenta, violet, and blue, respectively. Residues in the heavy chains of P5-22 Fab are yellow, while the light chains are orange, red, and green. (A) Structural superimposition of binary and ternary complexes. One steric clash between P14-44 from the binary complex and P5-22 in the ternary complex. Once P14-44 and P5-22 bind to one RBD simultaneously, these may disappear due to the flexibility of the loops between CH1 and the VH, LH, and VL domains. The structures of P14-44 Fab (B), the VH domain of P14-44 Fab (C), and the VL domain of P14-44 Fab (D) from binary to ternary complex (C).

Supplemental Figure 11 Comparison of epitopes of P5-22 Fab and P14-44 Fab to those of ACE2 and COVA1-16 on RBD in complex structures.

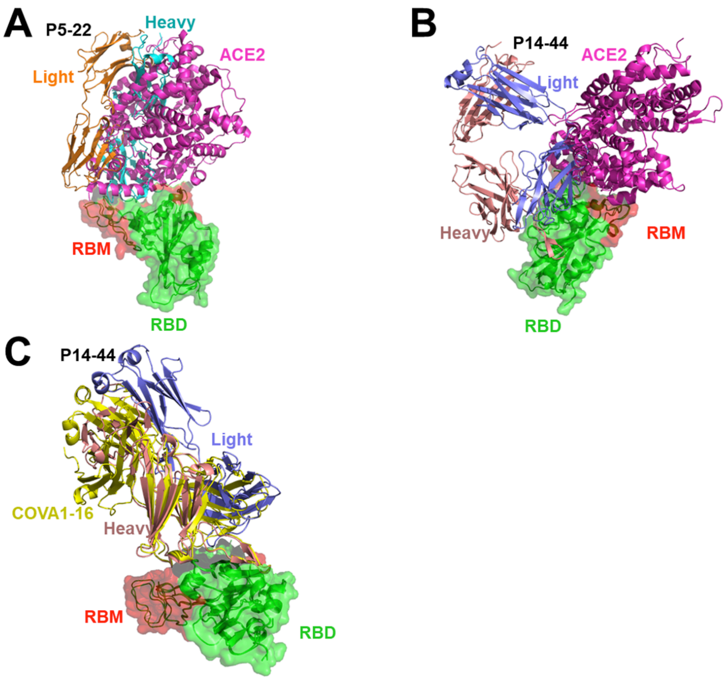

A
P5-22
Heavy
ACE2
Light
RBM
RBD
B
P14-44
ACE2
Light
Heavy
RBM
RBD
C
P14-44
Light
COVA1-16
Heavy
RBM
RBD

The secondary structure elements of ACE2 and COVA1-16 are colored in magenta and yellow, respectively. Residues in the heavy and light chains of P5-22 Fab and heavy and light chains of P14-44 Fab are colored in cyan, orange, blue, and violet, respectively. The surface of the RBD is colored green and that of the RBM in red. Comparison of epitopes of P5-22 (A), P14-44 (B), and ACE2 (PDB ID: 6M0J) on RBD. (C) Comparison of epitopes of P14-44 Fab to COVA1-16 (PDB ID: 7JMW) on RBD.

Supplemental Figure 12 P384 possesses a relatively close position to CDRH3 at P14-44.

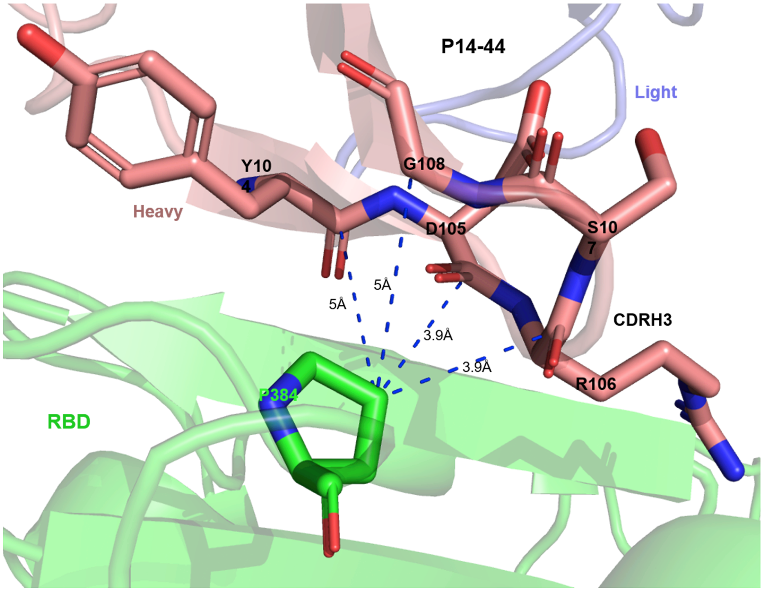

P14-44
Light
Heavy
Y10
4
G108
D105
S10
7
5Å
5Å
3.9Å
3.9Å
CDRH3
R106
P384
RBD

The secondary structure elements of the RBD. Heavy and light chains of P14-44 Fab are colored in green, blue, and violet, respectively. The residues of CDRH3 near P384 of the RBD are shown as sticks. Potential steric repulsions between CDRH3 and the sidechains of P384 mutants may be the reason why P14-44 is escaped by mutations at site P384.

Supplemental Figure 13 Sequence alignment of RBDs from selected coronavirus.

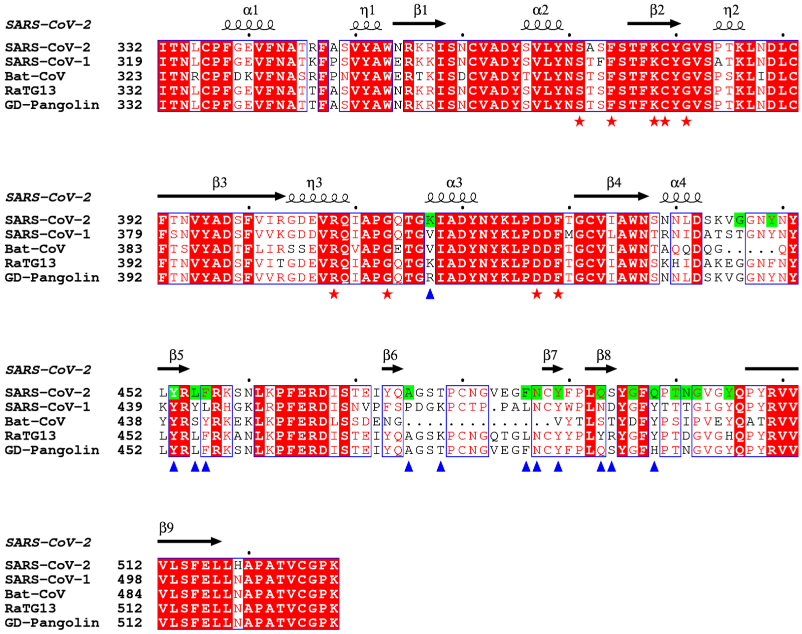
SARS-CoV-2
α1 η1 β1 α2 β2 η2
SARS-CoV-2 332 ITNLCPFGEVFNATRFASVYAWNRKRISNCVADYSVLYNSASFSTFKCYGVSPTKLNDLC
SARS-CoV-1 319 ITNLCPFGEVFNATKFPSVYAWERKKISNCVADYSVLYNSTFFSTFKCYGVSATKLNDLC
Bat-CoV 323 ITNRCPFDKVFNASRFPNVYAWERTKISDCVADYTVLYNSTSFSTFKCYGVSPSKLIDLC
RaTG13 332 ITNLCPFGEVFNATTFASVYAWNRKRISNCVADYSVLYNSTSFSTFKCYGVSPTKLNDLC
GD-Pangolin 332 ITNLCPFGEVFNATTFASVYAWNRKRISNCVADYSVLYNSTSFSTFKCYGVSPTKLNDLC
SARS-CoV-2
β3 η3 α3 β4 α4
SARS-CoV-2 392 FTNVYADSFVIRGDEVRQIAPGQTGKIADYNYKLPDDFTGCVIAWNSNNLDSKVGGNYNY
SARS-CoV-1 379 FSNVYADSFVVKGDDVRQIAPGQTGVIADYNYKLPDDFMGCVLAWNTRNIDATSTGNYNY
Bat-CoV 383 FTSVYADTFLIRSSEVRQVAPGETGVIADYNYKLPDDFTGCVIAWNTAQQDQG.....QY
RaTG13 392 FTNVYADSFVITGDEVRQIAPGQTGKIADYNYKLPDDFTGCVIAWNSKHIDAKEGGNFNY
GD-Pangolin 392 FTNVYADSFVVRGDEVRQIAPGQTGRIADYNYKLPDDFTGCVIAWNSNNLDSKVGGNYNY
SARS-CoV-2
β5 β6 β7 β8
SARS-CoV-2 452 LYRLFRKSNLKPFERDISTEIYQAGSTPCNGVEGFNCYFPLQSYGFQPTNGVGYQPYRVV
SARS-CoV-1 439 KYRYLRHGKLRPFERDISNVPFSPDGKPCTP.PALNCYWPLNDYGFYTTTGIGYQPYRVV
Bat-CoV 438 YYRSYRKEKLKPFERDLSSDENG..............VYTLSTYDFYPSIPVEYQATRVV
RaTG13 452 LYRLFRKANLKPFERDISTEIYQAGSKPCNGQTGLNCYYPLYRYGFYPTDGVGHQPYRVV
GD-Pangolin 452 LYRLFRKSNLKPFERDISTEIYQAGSTPCNGVEGFNCYFPLQSYGFHPTNGVGYQPYRVV
SARS-CoV-2
β9
SARS-CoV-2 512 VLSFELLHAPATVCGPK
SARS-CoV-1 498 VLSFELLNAPATVCGPK
Bat-CoV 484 VLSFELLNAPATVCGPK
RaTG13 512 VLSFELLNAPATVCGPK
GD-Pangolin 512 VLSFELLNAPATVCGPK

The sequences were aligned with the MUSCLE tool, and the figure was prepared using ESPript3.1. The sequences listed are for SARS-CoV-2, SARS-CoV-1, Bat-CoV, RaTG13, and GD-Pangolin. The second structural element of the RBD from SARS-CoV-2 is described above. Conserved residues are in red font, and identical residues are highlighted in red. The epitope residues of P5-22 and P14-44 on RBD are marked by blue triangles and red stars, respectively. Residues from RBD involved in ACE2 binding are highlighted in green.

Supplemental Figure 14 Polymeric REGN10933 and AZD8895 rescues RBD F486I binding but not F486R

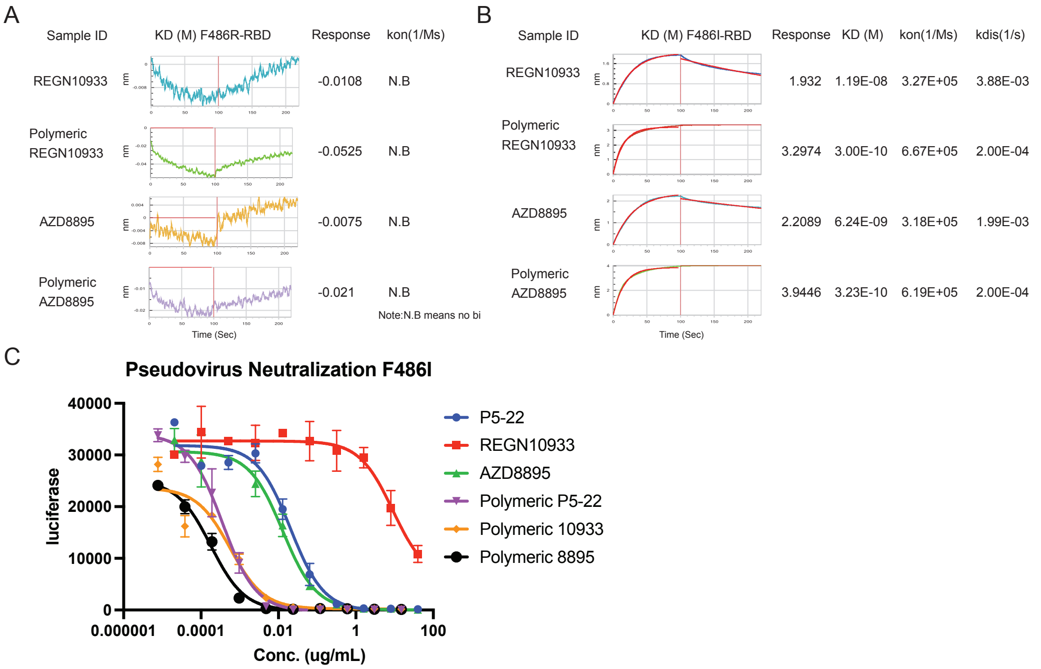

	P5-22	10933	AZD8895	Polymeric P5-22	Polymeric 10933	Polymeric 8895
IC50	0.02032	9.079	0.01284	0.0003575	0.0005692	0.0001798

(A,B) Affinities of indicated antibodies (100 nM) and RBD mutants (100 nM) were determined by biolayer interferometry (BLI) using ForteBio Red96e. Affinity data were analyzed using the ForteBio data analysis software version 10.0. (C) Representative neutralization curve of P5-22, REGN10933, AZD8895 and their corresponding polymeric IgGs in neutralizing F486I pseudovirus *in vitro.*

Supplemental Table 1: List of cloned S protein binders and their ELISA binding ODs and blocking capacities at 100 nM.

ELISA OD values for antibodies binding to SARS-CoV-1 and SARS-CoV-2 spike proteins, and the RBD protein. ELISA blocking assay to determine the percentage of blocking SARS-CoV-1 and SARS-CoV-2 RBD/hACE2 interaction when antibodies were at 100 nM.

Supplemental Table 2: Summary of 49 lead candidates

Epitope binning information for lead candidates, IC_{50} of pseudovirus neutralization, ELISA blocking of hACE2 and spike protein of SARS-CoV-2 and SARS-CoV-1, and antibody affinity information. Highlighted antibodies in deep green were selected as lead candidates for authentic

Supplemental Table 3: Complementarity-determining regions (CDR) and VDJ usage details of candidate antibodies.
