# Supplementary material for: Ultrapotent neutralizing antibodies against SARS-CoV-2 with a high degree of mutation resistance
Source: J Clin Invest. 2022 Feb 15;132(4):e154987. doi: 10.1172/JCI154987 (PMC8843702; doi:10.1172/JCI154987)
Supplement: Supplemental table 1 [file jci-132-154987-s013.pdf]

Ab(100nM)	Elisa Binding				Elisa blocking	
Clone ID	SARS-CoV-2 RBD	SARS-CoV-2 S	SARS-CoV-1 RBD	SARS-CoV-1 S	SARS-CoV-2 RBD	SARS-CoV-1 RBD
P7-8	4.2502	4.3763	0.0610	0.0464	95%	91%
P7-7	4.0483	3.8135	3.8903	4.1519	87%	97%
P7-21	4.3994	4.1488	0.2980	0.2028	7%	-3%
P7-19	4.3685	3.3817	0.0646	0.0459	-7%	10%
P7-15	1.2669	1.2404	1.4939	0.6100	98%	98%
P7-1	3.7043	4.3960	5.1859	3.6998	-40%	-29%
P5-8	0.2071	2.3619	N.D	3.3094	-12%	-14%
P5-66	4.4882	4.3759	N.D	0.0927	-80%	-8%
P5-65	3.9985	3.9110	0.1185	0.0549	98%	1%
P5-63	4.0161	3.9529	3.9959	4.1073	-3%	61%
P5-61	4.1937	3.8526	N.D	0.5276	-44%	-0%
P5-6	4.5471	3.9674	N.D	2.615	-31%	47%
P5-57	0.3511	1.2524	N.D	0.0858	14%	3%
P5-53	4.0525	4.0014	N.D	2.115	-39%	5%
P5-52	4.1561	4.1263	N.D	3.7775	-5%	-4%
P5-51	3.8747	3.9836	3.7675	4.0917	50%	86%
P5-50	4.0942	3.9896	N.D	3.7571	-8%	-5%
P5-5	3.9843	3.3390	3.3910	2.4949	97%	46%
P5-47	4.1587	4.4435	N.D	4.179	-82%	-146%
P5-45	4.257	4.1713	N.D	0.107	98%	-0%
P5-40	3.4371	3.6523	N.D	0.0694	96%	13%
P5-4	3.2688	2.0709	N.D	0.0629	58%	3%
P5-39	3.9490	3.4196	N.D	3.9737	-31%	20%
P5-38	3.9566	1.5454	N.D	0.0584	-63%	6%
P5-37	0.1614	3.0954	N.D	0.1387	-7%	-10%
P5-36	4.1778	4.3574	N.D	4.2905	82%	93%
P5-33	4.2338	4.6682	N.D	0.3447	98%	-7%
P5-32	4.1741	4.1885	N.D	4.2413	51%	88%
P5-31	3.2966	3.1508	N.D	2.9438	-73%	33%
P5-30	4.0466	3.7939	0.1501	0.1997	98%	-22%
P5-3	3.9827	4.0687	N.D	4.1369	31%	85%
P5-29	4.3694	2.7711	N.D	0.0701	-72%	-3%
P5-25	0.6596	0.5108	N.D	0.0933	-7%	-4%
P5-22	4.3081	4.2351	N.D	0.0899	98%	-4%
P5-21	2.5593	1.6318	0.2126	0.1441	-10%	-5%
P5-20	3.5828	1.8661	N.D	0.123	24%	21%
P5-2	4.2815	4.2986	N.D	2.2713	2%	54%
P5-17	2.9917	2.4429	N.D	0.383	92%	-3%
P5-16	4.2453	4.3335	N.D	0.0512	-60%	-0%
P5-15	0.3075	3.2954	0.0543	0.0506	-0%	-23%
P5-14	0.0971	3.6049	N.D	0.0609	5%	-10%
P5-13	3.9499	1.4558	2.0327	2.1970	5%	-24%
P5-12	4.309	4.3031	N.D	0.2815	-57%	0%
P5-1	0.1097	1.3021	N.D	0.0663	-1%	-1%
P3-8	0.0884	2.3654	0.0791	1.8635	22%	5%
P3-7	4.1317	4.0820	4.2850	4.0417	-14%	32%
P3-27	0.8506	0.8626	0.1217	0.0946	2%	5%
P3-26	0.0693	3.2693	0.0750	0.1024	-6%	-2%
P3-25	4.1636	4.0203	4.0936	3.9653	74%	91%
P3-21	4.1413	4.0276	4.0636	4.0483	-39%	-36%
P3-2	2.6028	2.3917	0.0691	0.0498	97%	22%
P3-17	4.1381	3.9267	0.1293	0.1354	94%	-3%
P3-12	0.1420	3.8206	0.1334	4.0188	-11%	-3%
P3-11	2.4426	2.7888	0.0602	0.0852	97%	4%
P3-10	4.1072	3.8822	3.9431	2.4242	38%	30%
P28-61	3.539	3.240	0.083	0.065	96%	-1%
P28-60	3.677	3.409	3.758	3.609	-8%	60%
P28-51	3.507	3.254	3.442	3.241	-13%	-4%
P28-46	0.123	3.040	0.098	3.218	2%	0%
P28-35	3.612	3.365	3.664	3.491	65%	92%
P28-34	0.074	2.885	0.083	0.078	-7%	-6%
P28-33	0.116	3.123	0.101	2.804	-6%	-6%
P28-32	3.352	3.344	3.604	3.325	26%	44%
P28-31	3.081	1.899	0.342	0.104	-23%	-0%
P28-30	2.997	3.307	0.109	0.088	95%	2%

P28-3	2.869	3.258	3.614	3.000	-39%	-25%
P28-27	3.625	3.215	3.656	3.277	52%	79%
P28-25	0.265	2.782	0.240	0.521	8%	-6%
P28-16	3.385	3.253	3.586	3.243	-47%	-40%
P28-15	3.500	3.241	3.593	2.966	-60%	-19%
P28-11	3.679	3.258	1.644	0.070	94%	-2%
P23-9	0.273	3.232	0.219	0.135	-18%	-37%
P23-7	2.053	2.976	2.284	2.698	-29%	-23%
P23-6	2.058	3.218	0.095	0.082	35%	12%
P23-37	3.081	2.640	0.126	0.086	67%	57%
P23-36	3.510	3.395	3.599	0.630	-21%	39%
P23-34	2.271	2.679	3.740	0.857	-100%	33%
P23-33	2.579	3.131	3.314	3.370	94%	92%
P23-30	2.022	3.493	3.107	3.342	-70%	-61%
P23-29	2.839	3.159	0.322	0.172	83%	-20%
P23-26	2.689	2.803	0.242	0.101	-51%	1%
P23-25	1.806	2.894	1.731	0.525	94%	17%
P23-19	3.488	3.867	3.039	3.383	-44%	15%
P23-16	0.073	3.728	0.081	2.121	-4%	-3%
P23-15	0.470	2.629	0.515	2.634	-10%	-27%
P23-12	0.094	3.259	0.098	0.085	-12%	-35%
P23-1	3.521	3.357	0.116	0.100	89%	59%
P21-9	1.046	2.924	0.730	0.520	-27%	-32%
P21-7	0.077	2.910	0.088	0.066	1%	-14%
P21-17	2.542	3.408	3.544	2.114	-14%	40%
P21-15	0.924	0.903	0.369	0.111	-5%	-9%
P21-12	2.235	2.884	3.229	3.232	93%	82%
P21-1	1.954	1.894	2.176	1.874	95%	95%
P1-9	0.0544	3.9975	0.0564	2.0244	98%	84%
P17-8	2.054	1.918	0.337	0.064	93%	-15%
P17-7	1.654	1.705	2.464	2.582	-35%	-7%
P17-6	0.195	2.097	0.133	1.282	-20%	-31%
P17-5	0.287	1.907	0.260	0.378	-20%	-31%
P17-4	2.036	1.951	3.046	2.669	-48%	-54%
P17-3	0.706	2.097	0.683	0.374	-6%	8%
P17-24	0.499	2.448	0.469	0.244	-5%	0%
P17-13	2.125	1.723	2.171	2.876	93%	95%
P17-10	3.191	2.406	0.107	0.084	95%	-2%
P17-1	0.134	1.899	0.110	0.307	-10%	0%
P16-9	0.0589	2.8610	0.0671	0.0691	5%	-1%
P16-8	0.0624	2.6898	0.0807	0.0630	9%	-2%
P16-7	0.0564	2.8706	0.0669	0.0475	3%	-8%
P16-5	0.0668	2.7903	0.0897	0.3659	3%	-7%
P16-4	0.0703	2.8042	0.0696	0.0876	39%	25%
P16-33	3.3546	3.1989	1.1396	0.0657	96%	16%
P16-30	3.0938	3.0159	0.2384	0.1000	97%	17%
P16-29	2.7171	2.3627	3.1954	2.3262	-53%	-5%
P16-23	2.9572	3.0269	2.8364	1.9680	-3%	8%
P16-22	0.0644	2.3019	0.0619	0.0465	15%	-0%
P16-21	0.1901	3.0833	0.1319	0.5408	2%	-3%
P16-20	3.3553	2.1888	0.0578	0.0473	95%	-4%
P16-18	0.0653	3.2482	0.0765	3.2581	1%	-4%
P16-15	2.9651	3.0675	0.0679	0.0563	97%	-2%
P16-14	0.0703	2.6885	0.0666	1.8287	27%	17%
P16-11	0.0644	2.8865	0.0812	0.0522	9%	-4%
P16-1	0.0623	2.6729	0.0618	0.0557	29%	23%
P15-9	3.4526	4.0610	0.5274	1.1141	-132%	-79%
P15-8	4.0364	3.9338	0.0694	0.0622	-304%	-86%
P15-6	3.8858	3.9283	3.5189	3.0455	-317%	-63%
P15-5	3.9665	3.8872	0.1518	0.0804	-409%	-299%
P15-34	3.8054	3.7718	0.0659	0.0607	90%	-38%
P15-32	0.1159	3.4958	0.0710	0.1147	-116%	-176%
P15-28	3.2345	3.7484	0.0641	0.0558	90%	-287%
P15-25	3.8775	3.6823	0.0703	0.0708	-412%	-303%
P15-24	3.9011	3.6230	0.0784	0.0608	-506%	-272%
P15-23	3.1792	3.9273	3.8987	3.9159	-96%	-12%
P15-22	3.8877	3.5469	0.0615	0.0516	89%	-27%
P15-20	4.0407	3.7150	1.8955	0.0921	-186%	-98%
P15-16	3.5466	3.9138	3.9895	4.0392	90%	86%
P15-15	0.0839	3.6319	0.0624	0.0523	-172%	-340%

P15-12	3.5878	3.9647	0.2241	0.0590	91%	1%
P15-1	3.8927	3.9292	3.7084	3.5267	-281%	-263%
P14-9	2.686	2.9828	2.9702	2.8044	-117%	6%
P14-53	3.8725	3.9155	0.0758	0.0597	93%	-22%
P14-46	3.6827	3.7503	0.1366	0.0910	92%	-15%
P14-45	3.9155	4.1354	4.0011	3.8582	-159%	-13%
P14-44	3.6573	3.9675	4.0181	3.9551	96%	95%
P14-43	3.5594	3.9756	3.2138	1.8407	-113%	14%
P14-4	0.3656	2.8611	0.1586	0.0767	13%	7%
P14-37	3.6978	3.2845	0.1488	0.0994	96%	-18%
P14-35	3.9243	3.9077	0.4375	0.0687	-78%	-22%
P14-30	3.4604	3.8251	4.1396	3.7949	-137%	-35%
P14-25	3.5656	3.7130	4.0130	3.7607	94%	77%
P14-23	0.1913	3.8354	0.1076	0.0656	-23%	-21%
P14-21	3.6828	2.5039	2.1849	0.2269	-8%	-15%
P14-20	2.4137	3.8654	4.0051	3.3396	96%	7%
P14-19	2.9434	3.7935	3.8571	3.6271	-163%	-17%
P14-16	0.1137	2.7224	0.1211	0.0604	-0%	-11%
P14-15	2.565	2.8906	3.0083	2.9503	-16%	80%
P14-13	3.8105	3.8657	0.7507	0.2519	-171%	-17%
P14-12	3.9961	4.0473	0.1124	0.0918	-200%	-19%
P14-1	2.4508	2.8777	0.1252	0.0547	45%	16%
P1-4	3.1960	2.1737	3.6194	4.1209	-190%	-97%
P13-4	0.0888	2.8144	0.1087	0.3931	9%	7%
P13-12	3.3802	3.0266	3.5067	2.8901	6%	71%
P12-9	0.070	2.294	0.072	0.058	-7%	-0%
P1-28	0.1309	3.8611	0.1326	0.1233	-10%	-19%
P1-23	0.1560	3.6941	0.1640	3.6185	0%	-3%
P1-22	0.1072	3.0702	0.1246	3.5378	-2%	-1%
P1-17	0.2300	3.6908	0.1856	0.1166	-8%	-3%
P10-8	0.0512	2.7697	0.0572	0.0407	-10%	-2%
P10-4	2.7633	2.8592	0.1092	0.0878	97%	-9%
P10-21	2.9104	2.9824	2.2597	1.2712	-72%	-2%
P10-20	2.8938	3.0155	0.0568	0.0409	97%	-4%
P10-16	2.7845	2.9171	0.0662	0.0455	-120%	8%
P10-12	0.0473	1.9893	0.0548	0.0393	27%	17%
P5-60	4.1073	0.4917	N.D	0.0693	2%	-4%
P14-8	0.5659	0.4847	0.1287	0.0748	13%	-0%
P10-11	0.5032	0.4672	0.0575	0.04	18%	3%
P23-17	0.413	0.419	0.127	0.114	-42%	-33%
P5-10	0.7358	0.3773	0.7735	0.2847	-7%	-26%
P17-9	0.838	0.353	0.644	0.484	-9%	-12%
P1-24	0.4752	0.3113	0.4389	0.3566	-12%	-14%
P7-23	0.4395	0.3064	0.1112	0.1285	-5%	4%
P3-13	0.5915	0.2986	0.5235	0.2804	-13%	-2%
P5-24	0.3649	0.2904	0.2104	0.2708	4%	-29%
P1-27	0.3008	0.2860	0.3595	0.3317	-13%	-11%
P17-20	0.295	0.223	0.197	0.135	-14%	-13%
P5-54	0.2442	0.2222	N.D	0.1033	6%	-2%
P1-26	0.3548	0.2189	0.3492	0.2312	-2%	12%
P3-22	0.2274	0.2040	0.1372	0.1103	-10%	-5%
P3-23	0.2126	0.1997	0.2305	0.1486	-8%	-6%
P5-9	0.306	0.1967	N.D	0.119	-1%	-12%
P17-19	0.209	0.185	0.291	0.096	-14%	-20%
P1-20	0.2009	0.1831	0.2614	0.1433	-10%	11%
P5-55	0.3313	0.1733	0.0836	0.0777	4%	-12%
P5-34	0.2086	0.1644	N.D	0.107	1%	-2%
P10-23	0.1815	0.1617	0.0543	0.0372	-1%	-6%
P10-6	0.137	0.1542	0.0736	0.053	-10%	19%
P1-18	0.2224	0.1433	0.1912	0.1134	-8%	-11%
P1-2	0.3328	0.1398	0.2510	0.1416	7%	3%
P17-15	0.124	0.138	0.172	0.109	-18%	-22%
P15-7	0.1687	0.1355	0.1030	0.0692	-139%	-180%
P15-18	0.1543	0.1353	0.1599	0.1070	-96%	-120%
P15-17	0.1585	0.1333	0.1312	0.0967	-129%	-186%

P5-42	0.2786	0.1317	0.1198	0.1356	14%	-10%
P17-21	0.166	0.131	0.134	0.086	-8%	-4%
P23-23	0.306	0.128	0.332	0.202	59%	57%
P3-4	0.1897	0.1269	0.1673	0.1158	3%	-3%
P1-19	0.1928	0.1234	0.1396	0.0801	-18%	-2%
P5-44	1.3104	0.1211	N.D	0.0586	13%	3%
P15-13	0.1042	0.1135	0.0726	0.0531	-170%	-295%
P3-6	0.1728	0.1119	0.1440	0.1091	-3%	-3%
P10-17	0.0485	0.111	0.0589	0.0459	9%	1%
P1-21	0.1939	0.1104	0.1507	0.1336	-1%	0%
P5-26	0.5604	0.1060	0.1744	0.1268	1%	-28%
P1-15	0.2386	0.0976	0.1758	0.1274	-17%	-11%
P3-20	0.1033	0.0970	0.0727	0.0603	-5%	-7%
P16-13	0.0973	0.0970	0.1030	0.0571	22%	10%
P5-41	0.0961	0.0954	0.0941	0.1252	10%	-27%
P1-5	0.2282	0.0951	0.1334	0.1332	-12%	1%
P3-14	0.1206	0.0951	0.0718	0.0528	2%	-0%
P3-24	0.1000	0.0890	0.1041	0.0870	-9%	-6%
P7-2	0.1230	0.0886	0.1518	0.0799	50%	17%
P28-5	0.112	0.088	0.097	0.072	4%	-3%
P28-7	0.105	0.081	0.296	0.077	1%	-5%
P23-39	0.101	0.078	0.089	0.065	24%	18%
P1-10	0.0848	0.0762	0.0747	0.0680	98%	97%
P13-13	0.0975	0.0722	0.1004	0.0576	16%	19%
P12-1	0.058	0.071	0.073	0.051	-24%	-20%
P10-9	0.1014	0.0664	0.0804	0.0543	9%	-8%
P5-62	0.4045	0.0648	0.0688	0.0760	1%	1%
P17-17	0.065	0.061	0.076	0.055	-45%	-24%
P15-33	0.0699	0.0609	0.0663	0.0552	-119%	-88%
P13-5	0.0688	0.0594	0.0774	0.0690	12%	4%
P5-46	0.4553	0.0588	0.4000	0.0655	13%	5%
P15-11	0.0585	0.0581	0.0698	0.0565	6%	-34%
P12-7	0.119	0.058	0.105	0.065	-13%	-9%
P10-28	0.0615	0.0545	0.0559	0.0402	32%	10%
P1-14	0.0512	0.0513	0.0713	0.0461	-14%	7%
P7-22	0.0516	0.0512	0.0704	0.0454	1%	11%
P13-2	0.0547	0.0493	0.0644	0.0487	5%	-2%
P13-1	0.0542	0.0485	0.0629	0.0435	15%	-3%
P10-19	0.0516	0.0485	0.0598	0.0438	-5%	2%
P12-4	0.082	0.048	0.076	0.061	-25%	-22%
