# Supplementary material for: Ultrapotent neutralizing antibodies against SARS-CoV-2 with a high degree of mutation resistance
Source: J Clin Invest. 2022 Feb 15;132(4):e154987. doi: 10.1172/JCI154987 (PMC8843702; doi:10.1172/JCI154987)
Supplement: Supplemental table 2 [file jci-132-154987-s014.pdf]

No.	Clone	Bin	Pseudovirus neutralization IC50 (nM)	Elisa Blocking (IC50)		KD (M) antibodies on sensor tip, SARS-CoV-2 S protein in solution				KD (M) antibodies on sensor tip, SARS-CoV-1 S protein in solution			
				SARS-CoV2-RBD	SARS-CoV-1 RBD	Response	KD (M)	kon(1/Ms)	kdis(1/s)	Response	KD (M)	kon(1/Ms)	kdis(1/s)
1	P3-25	2	6.4	33.05	0.2956	0.4586	3.85E-09	1.38E+05	5.31E-04	1.65E-01	5.73E-08	5.91E+04	3.39E-03
2	P5-36	1/2/3/4/6	30	3.796	1.78	0.2087	2.67E-09	7.49E+04	2.00E-04	4.12E-02	3.59E-09	5.57E+04	2.00E-04
3	P14-44	2	1.3	0.8625	0.3518	0.347	4.12E-09	1.24E+05	5.09E-04	6.75E-02	9.04E-08	2.29E+04	2.07E-03
4	P15-16	2	0.7	1.229	1.135	0.3855	2.08E-09	1.57E+05	3.26E-04	9.22E-02	1.80E-08	3.72E+04	6.69E-04
5	P21-12	2	30	0.5362	7.743	0.1412	1.13E-08	5.66E+04	6.38E-04	2.95E-02	4.79E-07	9.75E+03	4.67E-03
6	P21-1	1/2/4	7	1.39	0.4505	0.2925	2.99E-09	1.12E+05	3.35E-04	9.42E-02	1.70E-08	1.98E+05	3.37E-03
7	P23-33	2	6	1.289	0.7053	0.3619	1.57E-09	1.27E+05	2.00E-04	2.54E-01	4.96E-09	6.67E+04	3.31E-04
8	P14-25	2	16	1.107	4.601	0.3563	1.56E-09	1.28E+05	2.00E-04	5.64E-02	8.24E-09	2.43E+04	2.00E-04
9	P5-5	2	6	0.9491	98.09	0.6278	1.66E-08	2.11E+05	3.49E-03	1.47E-01	4.94E-08	3.37E+04	1.67E-03
10	P3-21	3/6	1.2	Non block	Non block	0.6327	9.57E-10	2.17E+05	2.08E-04	2.90E-01	3.30E-09	6.83E+04	2.25E-04
11	P5-16	3/6	1.3	Non block	Non block	0.9272	8.13E-10	2.46E+05	2.00E-04	-1.50E-03	N.B		
12	P5-39	6	32	Non block	Non block	0.2217	2.09E-09	9.55E+04	2.00E-04	3.67E-02	1.05E-07	6.28E+04	6.61E-03
13	P10-16	3	0.9	Non block	Non block	0.5014	3.85E-09	2.64E+05	1.02E-03	-1.23E-02	N.B		
14	P14-45	3/6	1	Non block	Non block	0.5477	1.14E-09	2.85E+05	3.25E-04	1.43E-01	1.10E-09	1.82E+05	2.00E-04
15	P15-23	3/6	1	Non block	Non block	0.5252	1.70E-09	1.95E+05	3.32E-04	2.01E-01	6.66E-09	5.65E+04	3.76E-04
16	P16-23	6	0.6	Non block	Non block	0.0418	2.72E-08	7.79E+04	2.12E-03	2.42E-01	1.82E-08	1.04E+05	1.89E-03
17	P23-26	3/6	1.1	Non block	Non block	0.3962	1.50E-09	1.33E+05	2.00E-04	-8.00E-04	N.B		
18	P23-19	6	0.9	Non block	Non block	0.3895	4.48E-09	1.61E+05	7.23E-04	1.41E-01	1.83E-08	1.39E+05	2.54E-03
19	P28-16	6	0.4	Non block	Non block	0.1005	8.33E-09	3.91E+04	3.25E-04	9.56E-02	3.73E-08	6.33E+04	2.36E-03
20	P3-2	1	0.2	1.551	Non block	0.4323	1.15E-09	1.73E+05	2.00E-04	-0.0031	N.B		
21	P3-11	1/2	0.2	1.097	Non block	0.3178	2.12E-09	9.44E+04	2.00E-04	-0.0145	N.B		
22	P3-17	1/2/4	2.5	1.456	Non block	0.3742	2.40E-09	1.41E+05	3.37E-04	0.0052	N.B		
23	P5-33	1/3	5	1.346	Non block	1.1415	5.91E-10	3.39E+05	2.00E-04	-0.0047	N.B		
24	P5-22	1	<0.1	1.053	Non block	1.0978	5.63E-10	3.55E+05	2.00E-04	0.0124	N.B		
25	P5-45	1/2/3/4/6	NA	1.251	Non block	0.0667	2.86E-09	6.99E+04	2.00E-04	0.0038	N.B		
26	P5-40	2	NA	1.231	Non block	0.0667	2.86E-09	6.99E+04	2.00E-04	0.0038	N.B		
27	P5-17	1/2	0.2	11.92	Non block	0.1068	2.05E-08	1.70E+05	3.49E-03	0.002	N.B		
28	P5-65	4	1.1	1.493	Non block	0.5288	1.78E-09	1.13E+05	2.00E-04	-0.0068	N.B		
30	P5-30	5	2.8	1.664	Non block	1.0794	5.93E-10	3.37E+05	2.00E-04	-0.0344	N.B		
31	P10-4	1/2	0.5	1.197	Non block	0.3964	1.40E-09	1.42E+05	2.00E-04	-0.027	N.B		
32	P10-20	1/2/3/5	0.2	0.9568	Non block	0.5326	1.92E-09	2.47E+05	4.75E-04	0.0115	N.B		
32	P14-20	2/6	NA	1.861	Non block	0.0865	5.94E-09	4.62E+04	2.74E-04	-0.0283	N.B		
33	P14-37	3/4	0.4	1.135	Non block	0.4607	6.36E-10	2.03E+05	1.29E-04	-0.0178	N.B		
34	P14-53	3/4	0.7	4.711	Non block	0.4562	3.88E-09	1.89E+05	7.32E-04	-0.0222	N.B		
35	P14-46	1/2	NA	0.6838	Non block	0.2266	1.98E-08	2.01E+05	3.98E-03	-0.0192	N.B		
36	P15-12	1/2	0.4	1.171	Non block	0.3842	2.30E-09	1.56E+05	3.58E-04		N.B		
37	P15-28	1/3	0.4	1.671	Non block	0.5786	1.97E-09	2.58E+05	5.08E-04		N.B		
38	P15-34	1/3	0.2	1.236	Non block	0.5122	9.21E-09	2.84E+05	2.62E-03		N.B		
39	P15-22	1/2	0.3	7.26	Non block	0.2212	1.47E-08	9.98E+04	1.47E-03		N.B		
40	P16-15	1	0.9	1.078	Non block	0.2429	1.74E-09	1.15E+05	2.00E-04	-0.0055	N.B		
41	P16-30	1	0.2	1.066	Non block	0.1728	3.17E-09	8.30E+04	2.63E-04	-0.0093	N.B		
42	P16-33	2	6.3	1.605	Non block	0.3082	1.59E-09	1.26E+05	2.00E-04	-0.0082	N.B		
43	P16-20	1/2/3	0.9	0.9414	Non block	0.3021	1.63E-09	1.23E+05	2.00E-04	-0.0123	N.B		
44	P23-29	1/2/3	0.2	1.196	Non block	0.2355	2.18E-09	9.17E+04	2.00E-04	-0.0053	N.B		
45	P23-6	3/6	1.6	47.78	Non block	0.5602	9.64E-10	2.07E+05	2.00E-04	-0.0138	N.B		
46	P23-1	3/6	NA	403.3	Non block	0.1919	2.28E-09	8.76E+04	2.00E-04	-0.011	N.B		
47	P23-25	5	5.7	1.403	Non block	0.1828	3.06E-09	6.54E+04	2.00E-04	-0.0061	N.B		
48	P28-30	1/2	3.8	1.062	Non block	0.2587	1.88E-09	1.07E+05	2.00E-04	-0.0019	N.B		
49	P28-61	1/2	0.6	1.033	Non block	0.153	2.56E-09	7.81E+04	2.00E-04	0.0056	N.B		
